# A review of neurophysiological relationships between sleep disorders and depression

**DOI:** 10.1016/j.bbih.2026.101171

**Published:** 2026-01-03

**Authors:** Yifan Huang

**Affiliations:** The ANU College of Science and Medicine, The Australian National University, Canberra, ACT, 2601, Australia

**Keywords:** Sleep disorders, Depression, Neurophysiology, HPA axis, Monoamines, Circadian rhythm, GABA

## Abstract

This review critically synthesizes current neurophysiological findings on the comorbidity between depression and sleep disorders. Drawing upon a multidisciplinary body of literature, the paper delineates overlapping neurochemical, hormonal, and inflammatory mechanisms. Further, it explores the role of astrocytic dysfunction and glutamate-GABA imbalance in reinforcing pathological feedback loops. By adopting an integrative framework, this review underscores the bidirectional and systemic nature of sleep disorder-depression comorbidity, offering insights into shared pathophysiological substrates and potential therapeutic targets for future research.

## Highlights

• The neurophysiological mechanisms of sleep-depression comorbidity are systematically reviewed.
• A framework integrating monoamine, HPA axis, and inflammatory factor dysregulation is proposed.
• Astrocytic dysfunction and GABA/glutamate imbalance are identified as key novel mechanisms.
• Clarifying these shared targets provides direct pathways for novel therapeutic development.
• Treating sleep disorders is posited as a viable strategy for improving depression outcomes.

## 1 Introduction

Sleep is a fundamental physiological process essential for physical health and psychological well-being. It is regulated by complex neural systems that coordinate transitions between wakefulness and sleep stages through intricate circuitry and neurotransmitter dynamics (Saper and Fuller, 2017). Sleep disorders encompass a range of conditions that disrupt normal sleep architecture, including obstructive sleep apnea, insomnia, reduced sleep duration, restless legs syndrome, and narcolepsy (Chen et al., 2014). These disorders may arise as primary conditions due to physiological dysfunction or maladaptive behaviors (Harvey, 2001) and are associated with adverse outcomes such as impaired glucose regulation, cardiovascular dysfunction, emotional dysregulation, and cognitive deficits (Amagai et al., 2010; Beihl et al., 2009; Chen et al., 2014; Itani et al., 2011).

Depression and sleep disorders exhibit a robust bidirectional relationship, and this association extends beyond insomnia to include other sleep disturbances such as hypersomnia, obstructive sleep apnea (OSA), and restless legs syndrome, all of which are significant prospective risk factors for depression (Germain & Kupfer, 2008; Kendzerska et al., 2014; Winkelman et al., 2008; Zhang et al., 2022). Among sleep disorders, insomnia is the most prevalent and is closely linked to depression. It serves not only as a symptom but also as a significant risk factor and early predictor of depressive episodes (Fernandez-Mendoza and Vgontzas, 2013; Otsuka et al., 2017). As evidenced by meta-analyses indicating that individuals with insomnia face a significantly increased risk of developing major depressive episodes (OR = 2.30) (Zhang et al., 2022). Besides, longitudinal studies indicate that insomnia increases the likelihood of developing depression (Baglioni et al., 2011), and its severity correlates with depressive symptoms (Riemann et al., 2001). Notably, treating insomnia can reduce depressive symptoms, suggesting a potential causal relationship (Manber et al., 2008).

Depression is a prevalent psychiatric disorder characterized by emotional, cognitive, and somatic symptoms, including persistent low mood, anhedonia, negative thoughts, cognitive impairment, appetite and sleep changes, fatigue, and physical pain (Harm et al., 2013; Malhi and Mann, 2018). Its lifetime prevalence is estimated at 11–17 % globally (Steinert et al., 2014), with peak vulnerability during adolescence, early adulthood, and late adulthood (Malhi et al., 2018). Neurobiologically, depression involves monoaminergic dysregulation, hypothalamic-pituitary-adrenal axis (HPA axis) hyperactivity, neuroinflammation, reduced neurotrophic signaling (e.g., serum brain-derived neurotrophic factor), and altered cortical–subcortical circuit function (Li et al., 2021; Plante, 2021). These mechanisms are also implicated in sleep regulation, suggesting shared neurophysiological foundations.

The pathophysiology underlying this comorbidity involves dysregulation of neuroplasticity, emotional processing, and the hypothalamic-pituitary-adrenal axis, with circadian rhythm disruptions playing a critical role (Drakatos et al., 2023; Liguori et al., 2021). Furthermore, specific patient populations, including those with Post COVID-19 syndrome and autoimmune diseases like primary Sjogren's syndrome, demonstrate a high prevalence of concurrent depression, anxiety, and sleep disorders, highlighting the shared mechanisms and substantial burden of these co-occurring conditions (Han et al., 2025; Seighali et al., 2024).

Recent research has identified potential biomarkers, such as decreased serum brain-derived neurotrophic factor (BDNF) levels, which are negatively correlated with poor sleep quality and demonstrate predictive value for sleep disorders in clinical populations (Han et al., 2025). The recognition of this strong interconnection necessitates integrated clinical approaches, including routine depression screening in sleep centers and sleep assessment in psychiatric settings, to facilitate early detection and tailored interventions (Daccò et al., 2025).

Despite extensive correlational data, few studies have systematically examined the neural substrates common to both conditions. This review addresses this gap by integrating neurophysiological research from EEG, neuroimaging, and neurochemical studies, with a focus on mechanistic insights underlying comorbidity.

## 2 Methods

This review was conducted as a narrative, mechanism-oriented synthesis and followed the SANRA (Scale for the Assessment of Narrative Review Articles) guidelines to ensure methodological transparency, coherence of scientific reasoning, and critical integration of heterogeneous evidence. Literature was identified through structured searches of PubMed, Embase, Web of Science, and the Australian National University Library databases, covering publications from 2000 to 2025. The literature search was restricted to studies published between 2000 and 2025 to ensure methodological consistency, clinical relevance, and alignment with contemporary research standards. Since 2000, substantial advances have occurred in diagnostic criteria for depression, sleep assessment techniques (e.g., standardized questionnaires and objective measures), and neurobiological and epidemiological methodologies. Earlier studies often relied on outdated diagnostic frameworks and less rigorous designs, which may limit their comparability with current evidence. Focusing on this time frame allows the review to capture modern theoretical models, robust longitudinal data, and clinically applicable findings, while older studies are considered primarily for historical context rather than as core evidence.

Search terms were used individually and in combination and included “sleep disorders,” “depression,” “comorbidity.” After reviewing additional literature, more keywords were employed for precise screening, including “circadian rhythm,” “GABA,” “glutamate,” “monoamines,” “HPA axis,” “inflammation,” and “astrocytes.” Studies were selected based on thematic relevance to neurophysiological mechanisms linking sleep disturbances and depression, rather than on uniform outcome measures.

See Appendix A Table 2 for an overview of selected studies. Inclusion criteria were: (1) studies published in English; (2) review articles focusing on either sleep disorders, depression, or both; (3) diagnosed depression using DSM-IV or DSM-5 criteria; (4) sleep disorders including insomnia, sleep apnea, circadian disorders, or restless legs syndrome; and (5) inclusion of neurophysiological measures such as EEG, fMRI, hormonal assays, or neurochemical markers.

The inclusion criteria for studies in Appendix B Table 3 were: (1) studies published in English; (2) human sample size ≥10; (3) original researches focusing on either sleep disorders, depression, or both; (4) inclusion of neurophysiological indicators such as circadian rhythms, neurotransmitters (e.g., GABA, serotonin, dopamine), neuroimaging, inflammatory markers, or brain network functions; and (5) exploration of the biological or neurophysiological mechanisms underlying the relationship between sleep and depression.

A standardized quality and risk-of-bias assessment was conducted for all included studies, and summary tables were generated to present the evaluations together with the principal potential sources of bias. Studies were classified by design type and assessed using established, design-specific instruments: animal experiments were evaluated with the SYRCLE risk-of-bias tool; observational studies (cohort, case–control, cross-sectional) with the Newcastle–Ottawa Scale (NOS); randomized controlled trials with the Cochrane Risk of Bias 2 (RoB 2) tool; and systematic reviews and meta-analyses with AMSTAR 2. Appendix C Table 4 provides a template summary table containing, for each reference, the study design, an overall quality or risk rating (or score where applicable), a domain-level evaluation (e.g., selection bias, information bias, confounding), and a concise description of the primary potential sources of bias (such as convenience sampling, inadequate blinding, small sample size, or incomplete adjustment for confounding variables). These standardized assessments were employed to support the interpretation of the findings and to inform the sensitivity analyses.

This review was conducted considering principles from the SANRA guideline. All studies were manually screened to ensure methodological validity and thematic relevance. Articles lacking clear neurophysiological focus, human applicability, or standardized diagnostic criteria were excluded. Priority was given to studies that integrated clinical findings with neurobiological mechanisms, thereby strengthening the analytical foundation for examining the bidirectional relationship between sleep disorders and depression.

## 3 Clarifying terminology

### 3.1 Sleep disorders vs. insomnia

While this review focuses on insomnia as a representative case, it is important to distinguish between the broader category of sleep disorders and specific diagnoses. Insomnia is characterized by difficulties in sleep initiation, maintenance, or early-morning awakenings, and is both a symptom and independent disorder. In contrast, other sleep disorders such as obstructive sleep apnea (linked to upper airway collapse and nocturnal hypoxia), restless legs syndrome (marked by limb discomfort and motor restlessness), and circadian rhythm disorders (involving misaligned sleep-wake cycles) manifest distinct pathophysiological and neurophysiological features. Each of these disorders may co-occur with depression and contribute to unique neurochemical imbalances.

### 3.2 SANRA vs. PRISMA

The SANRA framework was selected in preference to PRISMA because the primary objective of this review was to integrate and interpret diverse neurophysiological evidence rather than to conduct a systematic review or meta-analysis. PRISMA is optimized for quantitatively synthesizing homogeneous studies with comparable designs, populations, and outcome measures. In contrast, the present review encompasses heterogeneous methodologies, including epidemiological studies, neuroimaging, electrophysiology, molecular biomarkers, and preclinical animal research, which are not amenable to pooled statistical analysis. Consequently, formal meta-analytic procedures and fully reproducible search strategies were neither methodologically appropriate nor conceptually aligned with the aims of the study. SANRA provides a more suitable framework by emphasizing clarity of research rationale, transparency of literature selection, critical evaluation of evidence, and coherent mechanistic synthesis across disciplines, thereby supporting a comprehensive understanding of the shared neurophysiological substrates underlying the comorbidity between sleep disorders and depression.

### 3.3 Human vs. animal

In selecting literature from the database, both animal models and human clinical studies were systematically considered. Animal research provides crucial mechanistic insight into how sleep disturbances contribute to depression, allowing precise manipulation of genetic, environmental, and neurochemical factors to probe causal links among altered sleep architecture, circadian dysregulation, neuroinflammation, and depression-like behaviors (McEwen et al., 2015; Nollet et al., 2020). Such models facilitate controlled pharmacological or environmental interventions but cannot fully capture the heterogeneity, subjectivity, and species-specific sleep–circadian features of human depressive disorders, which limits translational applicability (LeGates et al., 2014; Nestler and Hyman, 2010).

In contrast, human epidemiological, longitudinal, and clinical studies provide direct ecological and clinical validity, documenting real-world insomnia, hypersomnia, circadian phase delay, and irregular rest–activity rhythms and their associations with depressive onset, severity, and treatment response (Baglioni et al., 2011; Fang et al., 2019). Technological advances, such as actigraphy, wearable monitoring, structured assessments, and multimodal functional measures, offer nuanced characterization of sleep–mood dynamics in daily life. Although these studies cannot experimentally manipulate underlying mechanisms, integrating clinical evidence with mechanistic insights from animal research is essential for linking preclinical hypotheses to clinically relevant models. Cross-species intermediate phenotypes and multimodal translational approaches thus remain central to clarifying how sleep and circadian disruption contribute to depression (Krause et al., 2017). Accordingly, this review treats human clinical research as primary evidence for shaping theoretical frameworks, with animal studies serving as complementary resources that enrich mechanistic understanding.

## 4 Sleep disorders

The etiology of sleep disorders is multifactorial, with insomnia being the most prevalent. This section examines contributing factors from two perspectives: lifestyle influences and comorbid medical conditions.

### 4.1 Lifestyle factors

Modern societal pressures, including chronic stress, alcohol consumption, and excessive internet use, are strongly associated with impaired sleep quality, typically manifesting as insomnia or insufficient sleep.

**Chronic Stress:** Chronic stress has emerged as a significant factor associated with sleep disturbances through both physiological and cognitive pathways. Empirical evidence indicates that stress not only correlates with objective sleep disruptions but also amplifies negative subjective perceptions of sleep via cognitive distortions (Alfasi and Soffer-Dudek, 2018). For instance, prolonged sleep latency, early awakenings, and parasomnias have been associated with a reduction in total sleep time of approximately 10 % (Otsuka et al., 2017). This bidirectional relationship may create a self-reinforcing cycle of stress, sleep disturbance, and psychological distress. Dysregulation of endocrine function (e.g., altered melatonin secretion) and maladaptive cognitive appraisal highlight the potential value of stress-reduction strategies in sleep health interventions. Longitudinal and interventional studies are warranted to confirm causal relationships.

**Alcohol Consumption:** Alcohol exerts complex effects on sleep. Earlier research suggested that moderate alcohol intake before bedtime might facilitate sleep onset (Johnson et al., 1998); however, subsequent studies have demonstrated that alcohol disrupts normal sleep architecture, producing altered sleep patterns. Consequently, alcohol is now considered a potentially detrimental sleep aid (Su et al., 2010). Acute intake may shorten sleep latency but impairs overall sleep quality, likely through modulation of cortical GABAergic systems and dopaminergic pathways (Koob and Colrain, 2020). Chronic use and subsequent abstinence-related sleep disturbances are associated with GABAA receptor desensitization, impaired dopaminergic signaling, hyperactivation of stress modulators (e.g., orexin, norepinephrine, CRF, and pro-inflammatory cytokines), and glutamatergic dysregulation. Future longitudinal studies are needed to clarify the temporal and causal dynamics.

**Internet Addiction:** Classified as a behavioral disorder, internet addiction is strongly associated with psychiatric conditions and sleep disturbances (Zhang et al., 2017). Excessive engagement with digital devices, including smartphones, computers, and televisions, may exacerbate stress and anxiety, contributing to sleep problems in both children and adults (Alfasi and Soffer-Dudek, 2018). Specific online activities, such as messaging and emailing, have been correlated with chronic stress and depressive symptoms, further increasing the risk of sleep disturbances (Thomée et al., 2011). Interventional studies are required to determine whether reducing internet use can causally improve sleep outcomes.

### 4.2 Comorbid medical conditions

Sleep disturbances associated with medical conditions are often less modifiable than those related to lifestyle factors. These include sleep impairments secondary to neurodegenerative diseases or other physiological conditions, as well as insomnia or narcolepsy triggered by disease episodes.

**Neurodegenerative Diseases:** Parkinson's disease (PD), a prototypical neurodegenerative disorder, is frequently associated with disruptions in sleep-wake regulation (Zuzuárregui and During, 2020). Pathological hallmarks involve progressive degeneration of neural circuits that regulate sleep-wake cycles, affecting neurotransmitter systems involved in sleep modulation, including noradrenergic, serotonergic, dopaminergic, and GABAergic pathways (Samizadeh et al., 2023). Imbalances in these systems have been linked to sleep disturbances, with common clinical manifestations including sleep fragmentation and reduced restorative sleep, characteristic features of insomnia (Chahine et al., 2017). Longitudinal studies are necessary to determine causal links between specific neurotransmitter dysregulation and sleep outcomes.

**Other Physiological Factors:** Chronic pain and nocturia are recognized as predisposing factors for sleep disturbances (Ito et al., 2020; Radziunas et al., 2018). These conditions disrupt sleep continuity through forced nocturnal arousals, leading to cumulative sleep deprivation and reduced sleep quality.

### 4.3 Neurophysiological mechanisms

Overall, dysregulation of sleep-regulating neurotransmitters and hormones, such as serotonin (Ito et al., 2020), melatonin (Prodhan et al., 2021), and dopamine (Samizadeh et al., 2023), represents a core neurophysiological basis for sleep disorders. While multiple factors contribute to sleep disturbances, neurological dysfunction appears central. Future interventional and longitudinal studies are essential to clarify causality and to develop targeted therapeutic strategies.

## 5 Depression

### 5.1 Etiology and risk factors

#### 5.1.1 Environmental factors

The pathogenesis of depression is widely recognized as involving a complex interplay between genetic predisposition and environmental influences. From an environmental perspective, chronic stress, early-life traumatic experiences (including physical abuse and emotional neglect), and dysfunctional family dynamics have been consistently associated with increased vulnerability to depression. These exposures are thought to interact with genetic background through mechanisms such as epigenetic modifications, thereby contributing to heightened risk of depressive disorders (Heim and Binder, 2012). The gene–environment interaction framework thus provides a useful theoretical basis for understanding the multifactorial etiology of depression.

Heim and Binder (2012) demonstrated that depression reflects the interaction of genetic susceptibility—including polymorphisms, familial psychiatric history, neuroendocrine characteristics, and personality traits—with adverse childhood experiences. Such pathogenic exposures are linked to enduring epigenetic alterations and sustained neurobiological dysregulation, including HPA axis hyperactivity, neurotransmitter imbalances, and structural as well as functional alterations in neural circuits. These maladaptive changes appear to increase susceptibility to depression and may also predispose individuals to comorbid medical conditions. Early interventions and comprehensive, multidisciplinary preventive approaches are therefore considered essential for reducing both individual suffering and the societal burden of depression. Longitudinal research is required to clarify the causal pathways linking specific environmental exposures to the onset of depressive disorders.

#### 5.1.2 Neurogenic basis

Recent evidence has increasingly emphasized the neurogenic basis of depression, expanding upon the classical monoamine hypothesis toward a neurotrophic–neurogenic model. This hypothesis posits that impaired adult hippocampal neurogenesis contributes to the pathophysiology of depression, particularly under chronic stress conditions. Eisch and Petrik (2012) reported that reduced proliferation and differentiation of neural progenitor cells in the dentate gyrus are associated with depressive-like behaviors, and that improvement of these deficits through antidepressant treatment is critical for symptom alleviation. Similarly, Lucassen et al. (2010) found that stress-induced dysregulation of the HPA axis is associated with impaired adult neurogenesis, correlating with depressive phenotypes.

Neurogenic biomarkers—including brain-derived neurotrophic factor (BDNF), Ki-67, doublecortin (DCX), and hippocampal volume—have shown consistent associations with depression severity. For example, Karege et al. (2002) observed significantly reduced serum BDNF levels in depressed patients compared with controls, with levels increasing after pharmacological treatment in parallel with reductions in Hamilton Depression Rating Scale (HAMD) scores. Structural MRI studies further demonstrate that reduced hippocampal volume, particularly in subfields such as CA1, CA3, and the dentate gyrus, is negatively correlated with depressive symptom severity (Huang et al., 2013). Mechanistically, BDNF signaling has been shown to promote neuroplasticity and stress resilience, suggesting that restoration of neurogenesis is both a mediator and an outcome of effective antidepressant treatment (Duman and Monteggia, 2006). Taken together, these findings support the potential of neurogenesis-related markers as both diagnostic indicators and therapeutic targets. Future interventional and longitudinal studies are necessary to determine whether impaired neurogenesis plays a causal role in depression or represents an associated consequence.

### 5.2 Neurobiological mechanisms

The pathophysiology of depression encompasses multiple hypotheses (Li et al., 2021), including: Monoamine hypothesis (Dysregulation of serotonin, norepinephrine, and dopamine); HPA axis dysfunction (Hyperactivity in stress-response pathways); Glutamatergic signaling (Excitotoxicity and synaptic plasticity deficits); GABAergic inhibition (Reduced GABA neurotransmission); Neurotrophic factors: (Impaired brain-derived neurotrophic factor signaling); Neuroinflammation (Elevated pro-inflammatory cytokines).

Furthermore, substantial evidence demonstrates mechanistic links between depression and sleep disorders. For example, Petit et al. (2021) identified significant associations between glucose metabolic dysregulation and both depressive symptoms and sleep regulation. On the other hand, Yang et al. (2023) revealed elevated levels of inflammatory biomarkers in individuals with either depression or insomnia, which shew that there may be shared pathophysiological pathways. Overall, these multidisciplinary studies demonstrate that sleep disorders and depression are not independent, unrelated conditions, but rather exhibit significant intersecting and overlapping relationships.

## 6 Neurophysiological analysis

### 6.1 Key neurobiological findings with effect size estimates

Table 1 summarizes the key neurobiological characteristics of sleep–depression comorbidity using standardized effect size metrics, providing multidimensional interpretive value. Reduced cortical GABA levels (d = −0.73) and elevated evening cortisol (OR = 1.52) demonstrate moderate-to-large effect sizes, suggesting GABAergic dysfunction and HPA axis hyperactivity as central features. Conversely, null findings for BDNF (p > 0.05) highlight limitations of the neurotrophic hypothesis in explaining comorbidity. Cross-system comparisons reveal that GABA dysregulation (d = −0.73) exerts a larger effect than inflammatory marker IL-8 (β = 0.30), indicating potential hierarchical contributions across neurobiological systems. These quantitative data provide an objective framework for understanding pathophysiology, inform clinical prioritization of GABA modulation and HPA axis monitoring, and underscore the need for future studies to examine cross-system interactions in greater detail.

**Table 1:** Key neurobiological findings with effect size estimates.

BiologicalSystem	Measure	EffectSize(95 %CI)	p-value	Study
GABAergic	Cortical GABA	d = −0.73 (−1.12, −0.34)	<0.001	Benson et al. (2020)
HPA Axis	Evening cortisol	OR = 1.52 (1.12, 2.06)	0.008	Pires Santiago et al. (2020)
Inflammation	IL-8 levels	β = 0.30 (0.15, 0.45)	0.001	Yang et al. (2023)
Neuroplasticity	BDNF	NS (p > 0.05)	–	Pires Santiago et al. (2020)

### 6.2 Pathological association mechanisms

Through analyses of clinical symptomatology and pathogenic mechanisms, a significant pathological association emerges between sleep disorders and depression. Neuroimaging and biochemical studies consistently support the role of neurophysiological mechanisms in the onset and progression of depression. Structural MRI investigations have demonstrated cortical thinning in prefrontal regions and reduced functional connectivity within the default mode network, while biochemical evidence highlights reduced serum BDNF levels, elevated cortisol concentrations, and alterations in REM latency as correlates of depression severity (Molendijk et al., 2014). Complementary EEG findings show a reduction in NREM stage 2 sleep duration (−22 min; p < 0.05) and shortened REM latency, reflecting HPA axis dysregulation and altered monoaminergic signaling (Plante, 2021; Wang et al., 2015).

At the therapeutic level, pharmacological evidence suggests a partial overlap in the pathogenic mechanisms of depression and sleep disorders. For instance, selective serotonin reuptake inhibitors (SSRIs) have been shown to alleviate both depressive symptoms and co-occurring sleep disturbances (Fava et al., 2006). As noted by Dhuna and Malkani (2020), these medications act primarily through monoaminergic neurotransmitter systems, which are integral to both affective regulation and the sleep–wake cycle. Emerging research continues to refine understanding of this neurobiological interdependence, emphasizing common neurotransmitter and neurohormonal substrates that link mood and sleep regulation.

Neuroimaging findings further substantiate these associations, revealing that patients with either insomnia or depression frequently exhibit similar neuroanatomical changes, including diminished cortical surface area in prefrontal regions (Plante, 2021). Such findings suggest that sleep disturbances may not simply represent secondary manifestations of depression but could serve as early neurobiological markers or prodromal features of mood disorders (Jackson et al., 2003). More granular analyses confirm significant cortical thinning in prefrontal areas among depressed individuals with sleep disturbances (mean reduction = 0.15 mm, 95 % CI: 0.12–0.18 mm, p < 0.001) (Sha et al., 2019). Parallel EEG investigations reveal consistent reductions in NREM stage 2 duration (mean = −22 min, SD = 5.2, p = 0.003) and shortened REM latency (20–30 % reduction, p < 0.01) (Plante, 2021). Nonetheless, these findings require cautious interpretation in light of inconsistent biomarker results. For example, Pires Santiago et al. (2020) reported non-significant BDNF changes, challenging straightforward neurotrophic explanations.

Taken together, current evidence suggests that sleep disorders and depression are linked by bidirectional pathophysiological mechanisms, with shared neurobiological substrates that extend beyond simple symptomatic overlap. Beyond these pathological correlations, substantial evidence implicates multiple neurochemical factors—including neurotransmitters and hormones—in the co-occurrence of sleep disturbances and depression, as systematically reviewed below.

### 6.3 Neurotransmitter systems

Neurotransmitters play a central role in the pathogenesis of both depression and primary insomnia, a relationship schematically illustrated in Fig. 2. Among these systems, GABA, serotonin (5-HT), dopamine, and norepinephrine are most consistently implicated. Dysfunction within these neurotransmitter networks provides a neurochemical basis for the frequent co-occurrence of depressive disorders and sleep disturbances.

GABAergic dysfunction has been strongly linked to both depression and sleep disorders (Benson et al., 2020). Reduced GABAergic activity has been observed in patients with insomnia and major depressive disorder, and sleep disturbances may further aggravate depressive symptoms by lowering central GABA levels. Conversely, depression can impair sleep regulation through overactivation of the HPA axis, which suppresses GABAergic inhibition (Hepsomali et al., 2020; Li et al., 2021; Luscher et al., 2011). This reciprocal interaction forms a self-perpetuating cycle in which sleep disruption and affective dysregulation reinforce one another.

Beyond GABA, serotonin and dopamine play critical roles in the regulation of wakefulness, sleep architecture, and affective stability. Serotonin exerts dual effects: while reduced serotonergic activity has been consistently associated with more severe depressive symptoms and higher suicidality risk (Luscher et al., 2011), excessive serotonergic signaling can increase REM sleep expression while disrupting sleep continuity, thereby degrading sleep quality (Alexandre et al., 2006; Monti and Jantos, 2008). Dopamine, by contrast, is central to reward and motivation circuits. Abnormal dopaminergic transmission has been linked to insomnia and depressive symptoms, in part through a feedforward loop that exacerbates dopamine system dysfunction (Finan and Smith, 2013). Such imbalances ultimately compromise both mood regulation and sleep stability.

Norepinephrine, another key monoaminergic neurotransmitter, exerts dual regulatory effects on emotional processing and sleep–wake control. Pharmacological evidence shows that monoamine reuptake inhibitors, which elevate extracellular norepinephrine and serotonin, suppress REM sleep while alleviating depressive symptoms (Wilson and Argyropoulos, 2005). However, the link between REM sleep abnormalities and depression is not entirely linear. More recent studies suggest that while monoaminergic systems regulate both REM sleep and affective states, the underlying neural pathways may be distinct but interconnected (Wang et al., 2012).

Taken together, these findings indicate that disturbances across GABAergic and monoaminergic systems contribute to both depression and primary insomnia. Neurotransmitter imbalances not only impair sleep quality but also destabilize mood, highlighting the shared neurochemical substrates that underpin their bidirectional relationship.

### 6.4 Hormonal regulation

Key hormonal factors, notably cortisol and melatonin, have been identified as significant biomarkers implicated in both sleep disorders and depression. These neuroendocrine markers have attracted considerable attention for their potential involvement in the shared pathophysiology of the two conditions. Fig. 3 illustrates this relationship: both disorders are associated with excessive activation of the HPA axis, abnormal cortisol secretion, and the emergence of a reinforcing cycle that links them. The following sections provide a detailed explanation.

Neurobiological studies suggest that hyperactivation of central cholinergic neurons may constitute a common neurobiological substrate for sleep disorders (e.g., shortened REM sleep latency) and depressive symptoms (e.g., dysregulated cortisol secretion rhythms) in individuals with depression (Poland et al., 1989). This dysfunction indicates that disruptions in cholinergic signaling are associated with downstream neuroendocrine imbalances, which may contribute to the amplification of pathological processes.

Subsequent research has highlighted the pivotal role of the HPA axis in both sleep–wake regulation and emotional modulation. Cortisol, a key stress hormone, plays a critical role in sleep onset and circadian rhythm maintenance. Dysregulation of the HPA axis is frequently observed in individuals with depression, and the severity of this dysregulation has been reported to correlate with stress exposure, depressive symptom intensity, and illness duration (Pires Santiago et al., 2020). These findings suggest that HPA axis dysfunction and abnormal cortisol secretion patterns may represent a central physiological link between sleep disturbances and major depressive disorder.

Melatonin, commonly referred to as the “sleep hormone,” not only regulates circadian rhythms but also contributes to the maintenance of healthy sleep patterns. Disruptions or reductions in melatonin secretion have been reported in individuals with depression. Consequently, therapeutic strategies such as light therapy, sleep deprivation therapy, and melatonin supplementation have been investigated for their potential to improve both depressive symptoms and sleep disturbances (Germain & Kupfer, 2008; Parry et al., 2019).

### 6.5 The immune-inflammatory system

A growing body of evidence indicates that aberrant activation of the immune–inflammatory system plays a central role in the pathophysiology of depression, while sleep disturbances further amplify depression risk via inflammatory pathways (Veler, 2023). Depressed individuals frequently exhibit elevated peripheral pro-inflammatory markers, including interleukin-1β (IL-1β), interleukin-6 (IL-6), tumor necrosis factor-α (TNF-α), and C-reactive protein (CRP) (Yang et al., 2023). These inflammatory signals can affect brain function through blood–brain barrier permeability, vagal afferent signaling, or microglial activation, inducing affective and cognitive changes characteristic of sickness behavior (Beurel et al., 2020; Dantzer et al., 2008). Sleep disturbances, particularly insomnia, show a robust bidirectional relationship with inflammation: sleep loss promotes systemic inflammatory activation, whereas inflammatory states disrupt sleep architecture and homeostasis, forming a self-reinforcing cycle (Irwin, 2019).

At the molecular level, pro-inflammatory cytokines (e.g., IL-6, TNF-α, IFN-γ) activate indoleamine 2,3-dioxygenase (IDO), diverting tryptophan metabolism from serotonin synthesis toward the kynurenine pathway, thereby reducing central serotonin availability and generating neuroactive metabolites such as quinolinic acid (Dantzer et al., 2008; Miller et al., 2009). In parallel, inflammation-induced microglial activation impairs synaptic integrity and neuroplasticity and disrupts circadian regulation via effects on the suprachiasmatic nucleus and melatonin rhythms (Irwin and Opp, 2017; Yirmiya et al., 2015). As Fig. 4 shown, together, these mechanisms provide a biological framework for depression–sleep disorder comorbidity and support integrated interventions targeting both inflammation and sleep dysfunction.

### 6.6 Additional mechanisms

Emerging evidence implicates the glutamate (Glu)/GABA system, and astrocytic metabolism in the pathophysiology of both sleep disorders and depression.

The Glu/GABA system plays a fundamental role in regulating both mood and sleep. Glutamate functions as the brain's primary excitatory neurotransmitter, while GABA—synthesized from glutamate—serves as the main inhibitory counterpart (Zhang et al., 2023). This excitatory–inhibitory balance is critical for normal brain function. In depression, evidence indicates that this balance is frequently disrupted, with excitatory and inhibitory signaling becoming misaligned (Chaves et al., 2018). In insomnia, shorter sleep duration has been hypothesized to associate with reduced glutamate levels (Benson et al., 2020). As depicted in Fig. 5, dysregulation of the Glu/GABA system likely represents a shared pathophysiological mechanism underlying both depression and sleep disorders.

Recent advances in neuroglial research have also highlighted the role of astrocytes in linking metabolism with neuropsychiatric conditions. Specific astrocytic subtypes regulate glucose metabolism in the central nervous system, contributing to neuroprotection, gliotransmission, and homeostatic stability (MacMahon Copas et al., 2021). Astrocytes are the primary sites for GABA uptake and play a key role in its synthesis (Zhang et al., 2023). Dysfunction in these astrocytic processes can disrupt brain energy balance and has been linked with mood and sleep disturbances (J.-M. Petit et al., 2021). As illustrated in Fig. 6, astrocytes influence these disorders indirectly, primarily through their regulation of neurotransmitter metabolism and neural energy supply.

## 7 Discussion

### 7.1 Overview

**Fig. 1:**
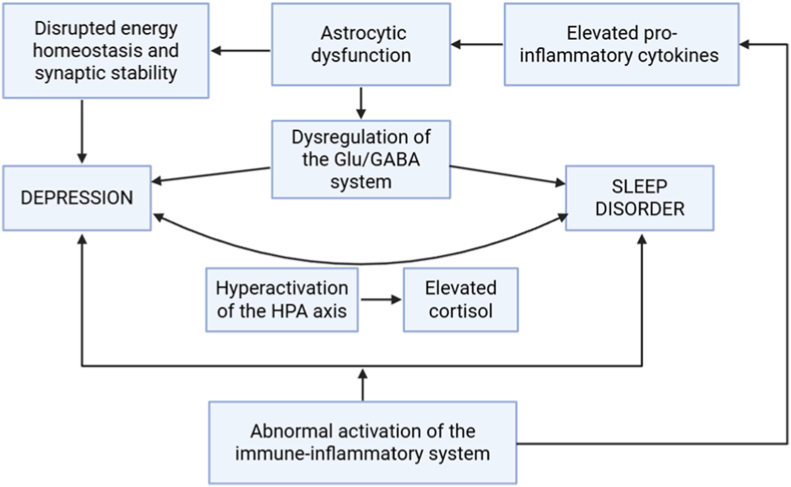
A simplified diagram of neurobiological substrates involved in sleep disorders and depression.

**Fig. 2:**
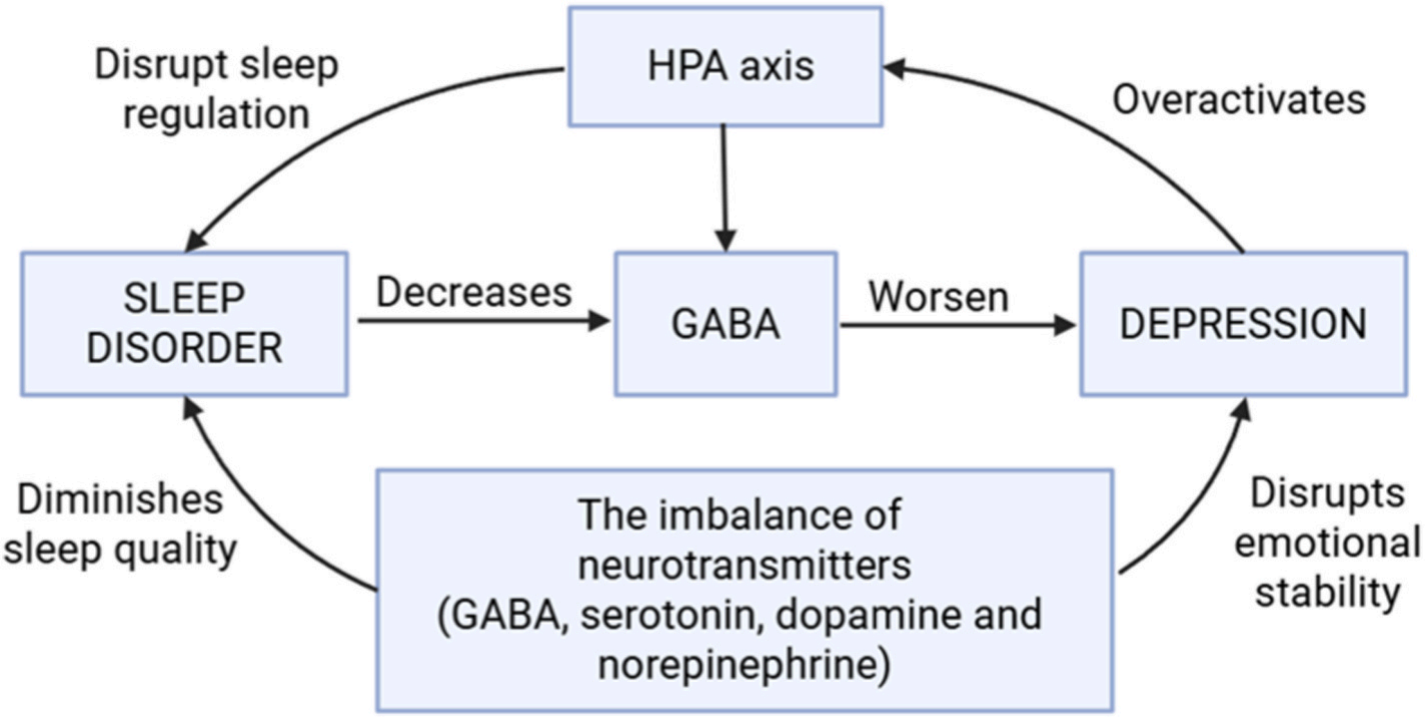
A Simplified Diagram of Neurotransmitter Systems Mechanisms Involved in Sleep Disorders and Depression.

**Fig. 3:**
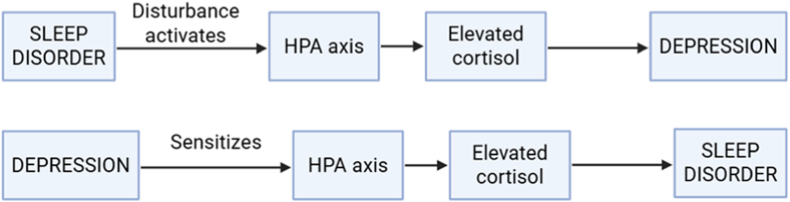
A Simplified Diagram of Hormonal Regulation Mechanisms Involved in Sleep Disorders and Depression.

**Fig. 4:**
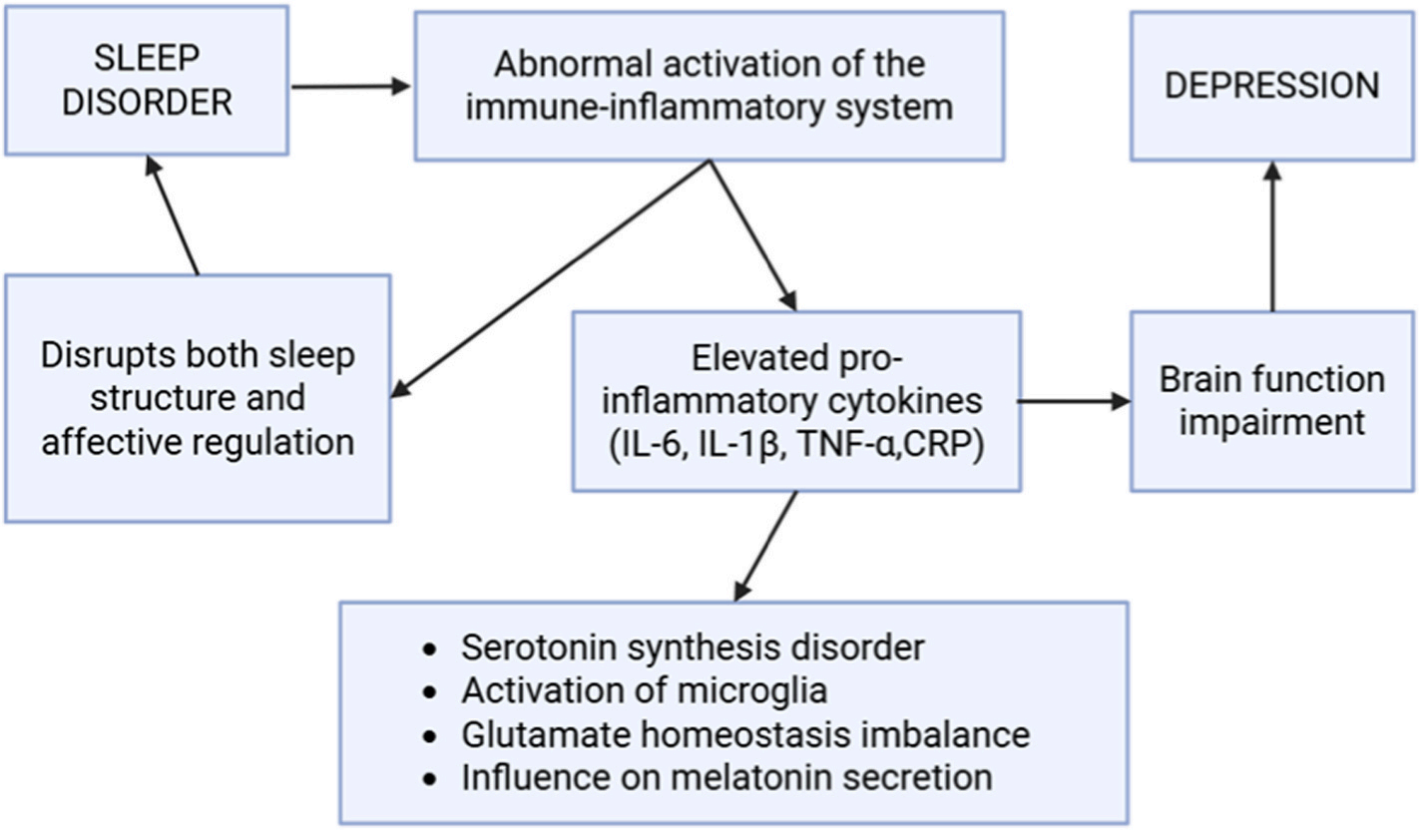
A Simplified Diagram of Inflammatory Factors Mechanisms Involved in Sleep Disorders and Depression.

**Fig. 5:**
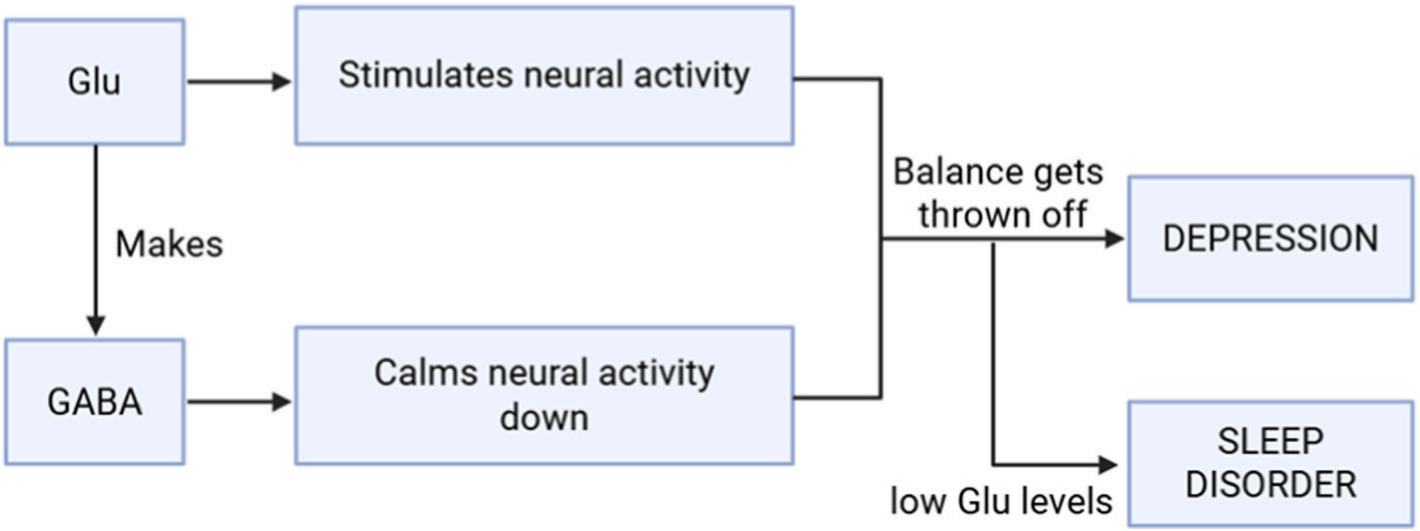
A Simplified Diagram of Glu/GABA Mechanisms Involved in Sleep Disorders and Depression.

**Fig. 6:**
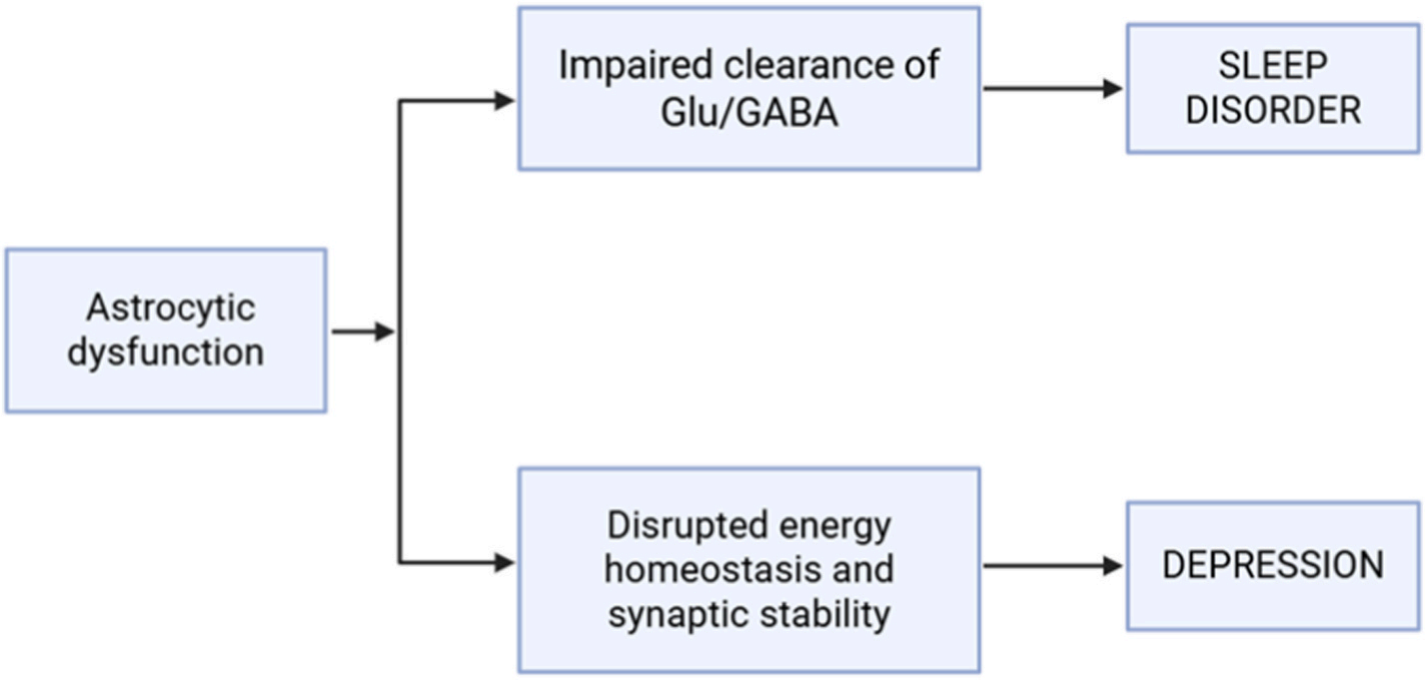
A Simplified Diagram of Astrocytic Function Involved in Sleep Disorders and Depression.

Sleep disturbances and depression are highly comorbid, with growing evidence suggesting a complex, bidirectional relationship. Rather than representing independent clinical phenomena, recent findings indicate that these conditions may share overlapping neurobiological substrates. Based on the aforementioned common theories and hypotheses, the neurobiological link between sleep disorders and depression is illustrated in Fig. 1.

Neuroimaging and electrophysiological studies consistently support this association. Structural MRI investigations have reported cortical thinning and reduced surface area in the prefrontal and anterior cingulate cortices among individuals with depression—regions also implicated in insomnia and impaired emotion regulation (Sha et al., 2019). Functional connectivity analyses further reveal disruptions within the default mode and salience networks across both disorders. Complementary EEG data demonstrate reduced non-REM stage 2 sleep duration (approximately −22 min; p < 0.05) and shortened REM sleep latency in depressed individuals, alterations linked to hyperactivation of the HPA axis and monoaminergic dysregulation (Plante, 2021).

Overlap in neurotransmitter dysfunction provides additional evidence of shared mechanisms. GABAergic deficits have been implicated in both insomnia and depression, with magnetic resonance spectroscopy studies reporting lower cortical GABA concentrations (Benson et al., 2020; Hepsomali et al., 2020). Antidepressants such as SSRIs have been shown to partially restore GABAergic tone while concurrently improving depressive and sleep-related symptoms (Nikhil A. Dhuna & Roneil G. Malkani, 2020). 5-HT, while essential for sleep initiation and mood stabilization, may also disrupt sleep continuity when present in excess, leading to increased REM density (Monti and Monti, 2007). Dysregulation of dopamine and norepinephrine further contributes to impaired reward processing and disrupted sleep–wake regulation. The HPA axis serves as a hormonal interface between sleep and affect, with elevated cortisol frequently observed in both acute insomnia and chronic depression. For instance, Pires Santiago et al. (2020) reported that the severity of HPA axis dysregulation correlates with illness duration and depressive symptom severity. Suppression of melatonin secretion, commonly observed in these conditions, has also motivated chronotherapeutic approaches such as bright light therapy and melatonin supplementation to address circadian and affective disturbances (Parry et al., 2019).

Beyond classical neurotransmitter models, emerging evidence highlights the contribution of inflammatory signaling, Glu/GABA imbalance, and astrocytic dysfunction to the co-occurrence of sleep disturbances and depression. Elevated pro-inflammatory cytokines, including IL-6 and TNF-α, have been consistently reported in individuals with poor sleep quality and depressive symptoms (Irwin and Opp, 2017). These inflammatory processes appear to influence both sleep architecture and mood regulation. Additionally, excitatory–inhibitory imbalance, characterized by altered glutamate and GABA transmission, has been associated with cortical hyperexcitability and emotional dysregulation (Chaves et al., 2018; Zhang et al., 2023). Astrocytes play a critical role in maintaining this balance by regulating glutamate clearance and GABA reuptake. Dysfunction in astrocytic metabolism has been linked to impaired energy homeostasis and excitatory–inhibitory imbalance, thereby increasing vulnerability to mood and sleep disorders (Hertz, 2004; J.-M. Petit et al., 2021). These findings collectively support a model in which overlapping neurochemical, hormonal, and glial mechanisms contribute to the frequent co-occurrence of insomnia and depression. However, longitudinal and interventional studies are required to confirm the causal pathways underlying these associations.

In summary, the co-occurrence of sleep disturbances and depression reflects multifactorial interactions encompassing structural and functional brain abnormalities, neurotransmitter imbalances, neuroendocrine dysregulation, and astrocytic dysfunction. These shared mechanisms underscore the importance of integrated diagnostic and therapeutic strategies that address mood and sleep disturbances simultaneously. Future research should emphasize longitudinal, mechanistic, and interventional designs to clarify causal pathways and inform precision medicine approaches.

### 7.2 Limitations

Despite the breadth and integrative scope of this review, several limitations warrant consideration.

(a) **Etiological ambiguity.** Extensive literature review reveals that, persistent epistemological and methodological limitations in contemporary research on the etiology of sleep disorders, which remains largely dominated by epidemiological designs and self-reported measures. Although such approaches have been valuable for identifying associations and population-level risk factors, they are inherently constrained to descriptive and correlational inference and offer limited insight into the neurobiological mechanisms that generate heterogeneous sleep pathologies. Insomnia, in particular, is frequently conceptualized as a unitary clinical entity, yet accumulating evidence suggests marked etiological heterogeneity: similar phenotypic manifestations may arise from fundamentally distinct neurophysiological processes. For example, delayed sleep onset resulting from circadian misalignment induced by artificial light exposure is unlikely to share identical mechanisms with sleep disruption secondary to systemic medical or inflammatory conditions, despite superficial clinical similarity. These pathways may differentially engage the HPA axis, melatonergic signaling, autonomic regulation, and affective neural circuits, underscoring the risk of mechanistic oversimplification when phenotypes are treated as homogeneous. Etiological ambiguity is even more pronounced in mood disorders such as depression, where sleep disturbance is both a core symptom and a prognostic factor. Although gene–environment interaction models dominate the field, they have yet to yield definitive causal accounts. Dysregulation of serotonergic transmission and sustained HPA axis hyperactivity are consistently observed in depressive states, but their causal status remains unresolved: it is unclear whether these alterations initiate depressive pathology or emerge as secondary adaptations to chronic stress and disturbed sleep. This uncertainty has substantial translational consequences. First-line pharmacotherapies, particularly selective serotonin reuptake inhibitors, largely provide symptomatic relief without clearly rectifying upstream biological perturbations, potentially explaining their variable efficacy and delayed onset of action. Addressing this gap requires a conceptual shift toward disentangling primary pathogenic mechanisms from compensatory neurochemical and neuroendocrine responses.
(b) **Publication Bias.** The current evidence base is highly vulnerable to publication bias. Positive findings demonstrating significant associations between sleep disturbances and depressive outcomes are disproportionately represented in the published literature, whereas null or negative studies are less likely to be reported. This bias may artificially inflate the strength of observed associations and foster premature causal inferences. Meta-analyses of insomnia and depression consistently show asymmetry suggestive of selective reporting (Baglioni et al., 2011; Doi et al., 2022). Future reviews should implement formal statistical assessments of publication bias (e.g., funnel plots, Egger's regression) and include unpublished or gray literature to mitigate overestimation of effect sizes.
(c) **Population Heterogeneity.** Most included studies inadequately account for demographic and sociocultural heterogeneity. Age, sex, cultural background, and socioeconomic status all influence both the prevalence and neurophysiological mechanisms of sleep–depression comorbidity. For instance, adolescence and late adulthood are peak vulnerability periods (Malhi et al., 2018), while sex differences in hormonal transitions (e.g., perinatal or menopausal states) strongly modulate risk (Parry et al., 2019). Moreover, cultural determinants such as chronotype variability and societal stress exposure shape both sleep and mood outcomes (Roenneberg and Merrow, 2016). The limited consideration of such moderators constrains generalizability and risks obscuring subgroup-specific pathways. Addressing these gaps will require stratified analyses, meta-regressions incorporating demographic moderators, and prospective multicenter cohorts across cultural contexts.
(d) **Translational Gap.** Another limitation concerns translational validity. Much molecular and neurophysiological evidence derives from animal models, particularly rodent paradigms of stress-induced insomnia or depressive-like behavior. While such models yield critical insights into HPA axis dysregulation and glutamatergic imbalance, extrapolation to humans is inherently constrained due to species differences in sleep architecture, immune signaling, and higher-order cognition (Kendler, 2019; Nestler and Hyman, 2010). Moreover, rodent paradigms often emphasize acute stress, failing to capture the chronic, multifactorial nature of human comorbidity. Bridging this gap requires more robust human studies, including longitudinal biomarker designs and clinical trials integrating mechanistic endpoints, to validate animal-derived hypotheses within clinically relevant contexts (Insel, 2014).
(e) **Insomnia Bias.** Finally, research disproportionately emphasizes insomnia relative to other sleep disorders. Although insomnia is highly prevalent and a strong predictor of depression, this narrow focus risks constraining theoretical models and clinical translation. Disorders such as obstructive sleep apnea, circadian rhythm sleep–wake disorders, and restless legs syndrome also show robust associations with depression but remain underexamined (Kendzerska et al., 2014; Winkelman et al., 2008). This insomnia-centric bias limits external validity. Future research should broaden the scope to include diverse sleep disorders, enabling comparative analyses and refining mechanistic frameworks.

**Future Directions for Addressing Limitations.** To overcome these limitations, several strategies are recommended. First, prospective meta-analyses should adopt bias-detection tools and incorporate gray literature to reduce publication bias. Second, primary studies should stratify analyses by age, sex, and socioeconomic status, and conduct cross-cultural investigations. Third, translational integration can be strengthened by aligning preclinical models with clinical endpoints and employing reverse-translation frameworks. Finally, expanding beyond insomnia through multicenter, cross-diagnostic cohorts will enhance generalizability. Together, these strategies will improve methodological rigor, strengthen causal inference, and guide more personalized and culturally sensitive interventions.

### 7.3 Proposed future research directions

Future research should prioritize large-scale, multicenter prospective cohort studies (target n > 500) with standardized multimodal assessments at baseline and follow-ups at 1 and 3 years. These assessments should include polysomnography (PSG), structural and functional MRI (high-resolution structural, resting-state connectivity, and optional task-based paradigms), and repeated serum and, when ethically permissible, cerebrospinal fluid sampling. Biomarkers should encompass HPA axis activity (free cortisol), inflammatory markers (IL-6, TNF-α, CRP), neurotrophic factors (BDNF), and indices of excitatory–inhibitory balance (MRS or plasma measures of Glu/Gln and GABA), complemented by astrocytic markers such as GFAP, S100B, and glycogen metabolism enzymes. Cohorts should systematically collect and adjust for major confounders (age, sex, BMI, lifestyle factors, comorbidities, and psychotropic/anti-inflammatory medication) and employ pre-registered analytic strategies with rigorous correction for multiple comparisons and transparent handling of missing data. Such multimodal, longitudinal designs are warranted by consistent epidemiological evidence linking sleep disturbances to increased risk of depressive episodes and allow mechanistic dissection of sleep–immune–neurotrophic–astrocytic pathways (Baglioni et al., 2011; Li et al., 2016; Wulff et al., 2010).

In parallel, stratified randomized controlled trials (RCTs) should be conducted to test both mechanistic and therapeutic hypotheses. Pharmacological mechanism trials should evaluate candidate compounds with astrocytic or metabolic targets (e.g., modulators of glycogenolysis, purinergic signaling, or glutamate clearance), with biomarker-based stratification and outcomes encompassing PSG-derived sleep continuity, sleep quality, depression severity (MADRS/HAM-D), inflammatory cytokines, BDNF, and imaging changes. Adjunctive sleep-targeted interventions (e.g., hypnotics or CBT-I combined with antidepressants) should be compared against antidepressant-only strategies in preregistered, blinded designs, with remission speed and stability as primary endpoints and biomarker shifts as secondary measures. To maximize reproducibility, standardized PSG, biospecimen, and MRI protocols are required, with resulting multimodal datasets enabling machine learning and causal inference approaches supported by external and cross-cultural validation. Data and biospecimen sharing under ethical frameworks will accelerate translation into risk predictors and companion diagnostics. Finally, given the temporal dynamics of sleep–mood interactions, future work should incorporate population-specific and chronotherapeutic approaches (e.g., timed wake therapy, light exposure) across developmental stages to identify individualized therapeutic windows, consistent with emerging evidence on sleep–inflammation mechanisms and chronotherapy (Depner et al., 2014; Irwin, 2019; Singh et al., 2021; Verkhratsky et al., 2021; Wu et al., 2015).

## 8 Conclusion

The intricate bidirectional interaction between sleep disorders and depression reflects a shared neurophysiological substrate involving neurotransmitter dysregulation, hormonal and circadian rhythm disruption, as well as neuroinflammatory processes. These converging mechanisms not only account for the high degree of comorbidity and symptom overlap between the two conditions but also offer promising targets for integrated therapeutic strategies. For example, these findings reinforce the hypothesis of shared neurobiological substrates in sleep disorders and depression. EEG and neuroimaging data reveal overlapping cortical abnormalities and HPA axis dysfunction. Pharmacological responses to SSRIs and melatonin-based therapies further confirm the co-involvement of monoaminergic and circadian systems. Nonetheless, the directionality remains underexplored. This framework advocates for a paradigm shift beyond traditional diagnostic boundaries, embracing a holistic approach to the treatment of comorbid psychiatric disorders. Progress in this field will require interdisciplinary collaboration and longitudinal, cross-cultural research designs to inform the development of more effective, personalized interventions that address dysfunctions in both sleep disorders and depression.

## Declaration of competing interest

I have nothing to declare.

## Figures and Tables

**Table 2:** Summary of Reviews Investigating the Neurophysiological Relationship Between Sleep Disorders and Depression

Author(s)	Article Type	Focus Area (Depression/Sleeep disorders)	Neural Indicator (s)	Main Findings
Harvey (2001)	Narrative review	Sleep disorders	Hyperarousal; cognitive-emotional arousal networks	Insomnia is conceptualized as an independent disorder rather than merely a symptom, involving persistent cognitive and physiological hyperarousal that overlaps with mood dysregulation.
Riemann et al. (2001)	Narrative review	Both	REM sleep alterations; HPA axis activity	Depression is associated with shortened REM latency, increased REM density, and dysregulated stress physiology, suggesting shared neurobiological substrates with sleep disturbance.
Ohayon (2002)	Epidemiological review	Sleep disorders	Population-level sleep parameters	Insomnia is highly prevalent and strongly associated with psychiatric disorders, especially depression, across cultures and age groups.
Jackson et al. (2003)	Systematic review	Depression	Prodromal affective and sleep symptoms	Sleep disturbances frequently precede both depressive and manic episodes, supporting their role as early warning markers.
Hertz (2004)	Narrative review	Both	Astrocyte–neuron lactate shuttle; astrocytic metabolism	Proposes astrocyte-derived lactate as a critical energy substrate for neurons, challenging neuron-centric models and highlighting glial involvement in brain function relevant to sleep and mood regulation.
Wilson and Argyropoulos (2005)	Qualitative review	Both	Monoaminergic systems (serotonin, norepinephrine)	Antidepressants differentially affect sleep architecture, with REM suppression and insomnia or hypersomnia as common side effects.
Duman and Monteggia (2006)	Narrative review	Depression	BDNF; synaptic plasticity	Introduces the neurotrophic hypothesis of depression, emphasizing stress-induced reductions in BDNF and synaptic connectivity as core mechanisms.
Monti and Monti (2007)	Narrative review	Sleep disorders	Dopaminergic pathways	Dopamine modulates wakefulness and REM sleep; dysregulation contributes to insomnia, hypersomnia, and mood disorders.
**WHO (2008)**	Global epidemiological report	Depression	Disability-adjusted life years (DALYs)	Depression is a leading global cause of disability, frequently comorbid with sleep disorders, amplifying disease burden.
Germain and Kupfer (2008)	Narrative review	Both	Circadian rhythm markers; REM sleep	Depression is characterized by circadian rhythm disruption and REM sleep abnormalities, implicating clock dysregulation in mood disorders.
Monti and Jantos (2008)	Narrative review	Sleep disorders	Dopamine and serotonin receptors	Dopaminergic and serotonergic systems jointly regulate sleep–wake states; imbalance contributes to insomnia and mood dysregulation.
Krishnan and Nestler (2008)	Narrative review	Depression	Stress signaling pathways; transcription factors	Depression arises from maladaptive molecular changes across limbic circuits driven by chronic stress.
Wulff et al. (2010)	Narrative review	Both	Circadian clock genes; SCN dysfunction	Circadian rhythm disruption is a core feature of psychiatric and neurodegenerative diseases, linking sleep dysregulation with mood pathology.
Nestler and Hyman (2010)	Conceptual review	Depression	Translational animal models	Evaluates strengths and limitations of animal models for understanding neuropsychiatric disorders, including depression and sleep phenotypes.
Lucassen et al. (2010)	Narrative review	Both	Adult hippocampal neurogenesis; inflammation	Stress, sleep disruption, and inflammation reduce neurogenesis, contributing to depression vulnerability and antidepressant mechanisms.
Baglioni et al. (2011)	Meta-analysis	Both	Longitudinal risk estimates	Insomnia significantly predicts future onset of depression, supporting a causal role of sleep disturbance.
Lüscher et al. (2011)	Narrative review	Depression	GABAergic interneurons	Major depressive disorder involves deficits in cortical GABAergic inhibition, altering emotional and cognitive regulation.
Weiss et al. (2011)	Clinical monograph	Sleep disorders	Nocturnal bladder–sleep interaction	Nocturia disrupts sleep continuity and contributes to reduced quality of life and mood symptoms.
Heim and Binder (2012)	Narrative review	Depression	HPA axis; epigenetic modifications	Early life stress alters stress-response systems via gene–environment interactions, increasing vulnerability to depression.
Eisch and Petrik (2012)	Narrative review	Depression	Hippocampal neurogenesis	Restoration of adult neurogenesis may be required for sustained antidepressant response.
Finan et al. (2013)	Narrative review	Sleep disorders	Pain–sleep regulatory circuits	Sleep disruption exacerbates pain sensitivity, suggesting bidirectional neurobiological interactions.
Finan and Smith (2013)	Narrative review	Both	Dopaminergic reward circuits	Insomnia, chronic pain, and depression share dopaminergic dysfunction as a potential unifying mechanism.
Kessler et al. (2013)	Epidemiological review	Depression	Cross-cultural prevalence metrics	Depression prevalence varies globally but consistently co-occurs with sleep problems across societies.
Vgontzas et al. (2013)	Narrative review	Sleep disorders	Objective short sleep duration; HPA activation	Insomnia with objectively short sleep represents the most biologically severe phenotype, with heightened cardiometabolic and psychiatric risk.
Fernandez-Mendoza and Vgontzas (2013)	Narrative review	Sleep disorders	HPA axis; autonomic activation	Chronic insomnia is linked to physiological hyperarousal and increased risk of mental and physical illness.
LeGates et al. (2014)	Narrative review	Both	Retinal–SCN pathways; melanopsin	Light exposure critically modulates circadian rhythms, sleep, and affective regulation.
Molendijk et al. (2014)	Systematic review & meta-analysis	Depression	Serum BDNF	Reduced peripheral BDNF levels are consistently associated with depression, supporting its role as a biomarker.
Steinert et al. (2014)	Systematic review	Depression	Long-term clinical course indicators	Adult depression often follows a chronic or recurrent course, with sleep disturbance predicting poorer outcomes.
Depner et al. (2014)	Narrative review	Sleep disorders	Circadian metabolic regulation	Sleep and circadian misalignment impair glucose metabolism and energy balance, indirectly affecting mood.
Insel (2014)	Conceptual framework	Depression	RDoC neural domains	Advocates dimensional, circuit-based approaches to psychiatric disorders beyond traditional diagnostic categories.
Wu et al. (2015)	Meta-analysis	Sleep disorders	Cognitive–behavioral regulation	CBT-I is effective for insomnia comorbid with psychiatric and medical disorders, including depression.
Polyakova et al. (2015)	Meta-analysis	Depression	BDNF	Increases in BDNF are associated with successful antidepressant treatment.
McEwen et al. (2015)	Narrative review	Depression	Allostatic load; stress hormones	Chronic stress reshapes brain structure and function, increasing susceptibility to depression.
Miller and Hen (2015)	Narrative review	Depression	Adult neurogenesis	Updates the neurogenic theory, integrating anxiety and stress-related mechanisms.
Wang et al. (2015)	Narrative review	Both	REM sleep circuitry; monoamines	REM sleep disturbances in depression reflect neurotransmitter imbalance and altered limbic activity.
Li et al. (2016)	Meta-analysis	Both	Longitudinal risk estimates	Insomnia significantly increases the risk of developing depression in prospective cohort studies.
Roenneberg and Merrow (2016)	Narrative review	Sleep disorders	Circadian clock genes	Misalignment between biological and social clocks adversely affects mental health.
**Kohyama(2016)**	Hypothesis article	Sleep disorders	Arousal networks	Proposes shared neuronal mechanisms underlying sleep disturbance in ASD and ADHD.
Anacker & Hen (2017)	Narrative review	Depression	Adult hippocampal neurogenesis	Reduced neurogenesis impairs cognitive flexibility and mood regulation, linking memory and depression.
Chahine et al. (2017)	Systematic review	Sleep disorders	Sleep–wake circuitry in Parkinson's disease	Sleep and circadian disorders are highly prevalent in Parkinson's disease and contribute to non-motor symptom burden.
Krause et al. (2017)	Narrative review	Sleep disorders	Prefrontal–limbic connectivity	Sleep deprivation impairs emotional regulation and cognitive control via altered brain network connectivity.
Saper and Fuller (2017)	Narrative review	Sleep disorders	Hypothalamic sleep–wake circuits	Wake–sleep regulation depends on integrated hypothalamic and brainstem networks.
De Aquino et al. (2018)	Narrative review	Depression	Epidemiological risk factors	Major depressive disorder shows cultural variability but consistent associations with stress and sleep disturbance.
Kishi et al. (2018)	Meta-analysis	Depression	BDNF	Confirms reduced BDNF levels in major depressive disorder across studies.
Malhi and Mann (2018)	Narrative review	Depression	Multilevel neural systems	Provides an integrated clinical and neurobiological overview of depression.
Malhi et al. (2018)	Clinical guideline	Depression	Treatment response markers	Evidence-based clinical recommendations for managing major depressive disorder.
Irwin (2019)	Narrative review	Both	Pro-inflammatory cytokines (IL-6, TNF-α)	Sleep and inflammation are bidirectionally linked, contributing to depression and systemic illness.
Szabo et al. (2019)	Narrative review	Sleep disorders	Hypocretin/orexin system; immune markers	Narcolepsy involves neuroimmune mechanisms affecting sleep–wake stability.
Fang et al. (2019)	Narrative review	Both	Neurotransmitters; HPA axis; inflammation	Sleep disturbance and depression show a bidirectional relationship mediated by neuroendocrine and inflammatory pathways.
Sha et al. (2019)	Meta-analytic neuroimaging study	Depression	Large-scale brain networks	Psychiatric disorders share common disruptions in cognitive–affective networks.
Kendler (2019)	Conceptual review	Depression	Etiological complexity	Psychiatric illness arises from multiple interacting causes rather than single mechanisms.
Irwin (2019)	Narrative review	Both	Pro-inflammatory cytokines	Sleep and inflammation interact bidirectionally, influencing depression risk and progression.
Hepsomali et al. (2020)	Systematic review	Sleep disorders	GABAergic signaling	Oral GABA supplementation shows modest benefits for stress and sleep in humans.
Nollet et al. (2020)	Narrative review	Both	Stress–sleep feedback loops	Sleep deprivation and stress reinforce each other, exacerbating mood disorders.
Borentain et al. (2020)	Observational study	Depression	Patient-reported outcomes	Real-world data show severe symptom burden in depression with suicidal ideation.
Dhuna and Malkani (2020)	Narrative review	Both	Neurotransmitter systems	Antidepressants variably affect sleep architecture, with clinical implications.
Koob and Colrain (2020)	Narrative review	Both	Allostatic stress systems	Alcohol use disorder and sleep disturbances form a feed-forward pathological cycle.
Zuzuárregui and During (2020)	Narrative review	Sleep disorders	Dopaminergic degeneration	Sleep disorders are common in Parkinson's disease and worsen non-motor symptoms.
**Wang et al. (2020)**	Experimental review	Both	Circadian genes; MAPK pathway	Rapid antidepressant effects may involve circadian gene regulation via MAPK signaling.
Petit et al., 2021, Petit et al., 2021	Narrative review	Both	Brain glycogen metabolism	Altered astrocytic glycogen metabolism may link sleep disturbance, headache, and depression.
Castrén & Monteggia (2021)	Narrative review	Depression	BDNF–TrkB signaling	Antidepressants enhance synaptic plasticity through BDNF-dependent mechanisms.
Plante (2021)	Narrative review	Both	Sleep–mood interaction pathways	Sleep disturbance is both a risk factor and maintaining mechanism for depression.
Prodhan et al. (2021)	Narrative review	Sleep disorders	Melatonin signaling	Melatonin dysregulation contributes to sleep disturbance in Alzheimer's disease.
Verkhratsky et al. (2021)	Narrative review	Both	Astrocytic dysfunction	Astrogliopathology plays a critical role in psychiatric disorders including depression.
Li et al. (2021)	Narrative review	Depression	Translational neuroscience	Reviews advances in neural mechanisms and treatment targets in major depressive disorder.
Zhang et al. (2022)	Systematic review & meta-analysis	Both	Longitudinal risk ratios	Sleep and circadian disorders significantly predict future depression.
Doi et al. (2022)	Methodological commentary	Depression	Risk estimation metrics	Questions overreliance on relative risk, relevant for psychiatric epidemiology.
Li et al. (2022)	Narrative review (animal models)	Both	Sleep–depression circuitry	Animal and primate models demonstrate shared biological substrates of sleep disturbance and depression.
**Xiao et al. (2022)**	Narrative review	Sleep disorders	Astrocytes; microglia	Glial pathology links sleep disturbance with Alzheimer's disease progression.
Samizadeh et al. (2023)	Narrative review	Sleep disorders	Melatonin; dopaminergic pathways	Parkinson's-related sleep disorders involve molecular disruptions in melatonin and dopamine systems.
**Mirchandaney et al. (2023)**	Narrative review	Both	Emerging neural mechanisms	Summarizes recent mechanistic advances in sleep–depression research.
Veler (2023)	Narrative review	Both	Inflammatory mediators	Reinforces the bidirectional relationship between sleep disturbance and inflammation.
Lim et al. (2023)	Policy review	Sleep disorders	Population sleep health metrics	Advocates integrating sleep health into global public health strategies.
Palmer et al. (2024)	Systematic review & meta-analysis	Both	Emotion-regulation neural networks	Experimental sleep loss robustly impairs emotional processing across decades of research.
Seighali et al. (2024)	Systematic review & meta-analysis	Both	Prevalence estimates	Depression and sleep disorders are highly prevalent among long-COVID patients, indicating persistent neuropsychiatric impact.

**Table 3:** Summary of original researches investigating the neurophysiological relationship between sleep disorders and depression

Author(s)	Sample Size	Design	Population	Sleep Disorder/Assessment
Karege et al. (2002)	31 MDD patients, 31 controls	Cross-sectional	Adults with major depression	N/A
Dew et al. (2003)	822	Prospective cohort	Healthy older adults	Sleep quality assessed via questionnaires and actigraphy
Lemonde et al. (2003)	92 MDD patients, 292 controls	Genetic association study	Adults with major depression and suicide history	N/A
Fava et al. (2006)	33	Randomized controlled trial	Adults with MDD and insomnia	Polysomnography (PSG) and sleep diaries
Field et al. (2007)	60 depressed pregnant women	Observational cohort	Pregnant women with depression	Actigraphy, sleep questionnaires
Manber et al. (2008)	60	RCT	Adults with MDD and insomnia	PSG, sleep diaries
Winkelman et al. (2008)	6000+	Cross-sectional	Adults from Sleep Heart Health Study	Restless legs syndrome assessed via clinical criteria
Beihl et al. (2009)	5102	Prospective cohort	Multiethnic adults	Self-reported sleep duration
Amagai et al. (2010)	11,367	Prospective cohort	Japanese adults	Self-reported sleep duration
Dregan and Armstrong (2010)	5032	Longitudinal cohort	Adolescents followed into adulthood	Self-reported sleep disturbances
Gass et al. (2010)	1189	Genetic association study	Adults with/without depression	Sleep disturbances assessed via questionnaires
Troxel et al. (2010)	1504	Prospective cohort	Adults	Sleep symptoms via questionnaires
Itani et al. (2011)	5847	Cross-sectional	Japanese adults	Sleep duration, shift work via questionnaires
Krause et al. (2017)	1417	Cross-sectional	Sleep clinic patients	Sleep disturbances via clinical assessment
Penninx et al. (2011)	2981	Prospective cohort	Adults with depression/anxiety	Sleep assessed via questionnaires
Thomée et al. (2011)	415	Prospective cohort	Young adults	Sleep disturbances via questionnaires
Batterham et al. (2012)	1469	Prospective cohort	Adults	Sleep disturbance via questionnaires
Giannaki et al. (2013)	58	RCT (partially double-blind)	Patients with uremic restless legs syndrome	Restless legs syndrome severity scales
Huang et al. (2013)	41 MDD patients, 43 controls	Cross-sectional	Adults with MDD	N/A
Winkelman et al. (2008)	22	Cross-sectional	Adults with REM sleep behavior disorder (RBD) comorbid with depression	REM sleep behavior disorder via PSG
Basch et al. (2014)	4 nationally representative samples	Cross-sectional	American high school students	Sleep duration via self-report
Chen et al. (2014)	3276	Cross-sectional (NHANES, 2005–2008)	US adults	Sleep apnea, insomnia, short sleep duration via questionnaires/clinical assessment
Kendzerska et al. (2014)	10,149	Historical cohort	Adults with obstructive sleep apnea	OSA via clinical diagnosis/PSG
Liu et al. (2016)	12,810	Cross-sectional (national survey)	US adults	Self-reported healthy sleep duration
Davari-Tanha et al. (2016)	96	RCT, double-blind, placebo-controlled	Menopausal women	Sleep disturbances via questionnaires
Parry et al. (2019)	56	Experimental/clinical trial	Pregnant and postpartum women	Sleep timing via PSG, melatonin assays
Zhang et al. (2017)	1058	Cross-sectional	Vietnamese youths	Sleep quality via PSQI
Hafner et al. (2017)	N/A	Cross-country comparative analysis	Adults	Sleep insufficiency assessed via surveys
Marques et al. (2017)	1162	Cross-sectional	Higher education students	Sleep quality via PSQI
Otsuka et al. (2017)	2293	Cross-sectional	Japanese adults	Sleep disorders via questionnaires
Alfasi and Soffer-Dudek (2018)	198	Cross-sectional	Adults	General sleep experiences via questionnaires
Burke et al. (2018)	1152	Cross-sectional	Older adults	Sleep disturbance via questionnaires
Radziunas et al. (2018)	65	Cross-sectional	Parkinson's disease patients	Sleep disturbances via questionnaires
Benson et al. (2020)	52	Cross-sectional	Adults with insomnia and MDD	Sleep quality via PSQI
Liguori et al. (2021)	28	Cross-sectional	Adults with idiopathic REM sleep behavior disorder	Actigraphy and PSG
Singh et al. (2021)	N/A	Commentary	Youth	N/A
Cai et al. (2022)	27,466	Cross-sectional (NHANES, 2005–2018)	US adults	Trouble sleeping via self-report
Li et al. (2022)	10,642	Cross-sectional (NHANES, 2007–2014)	US adults	Sleep duration and quality via questionnaires
Zhang et al. (2022)	1234	Cross-sectional	University students	Sleep quality via PSQI
Drakatos et al. (2023)	348	Cross-sectional/clinical cohort	Adults with recurrent depressive or bipolar disorder	PSG and questionnaires
Nikolic et al. (2023)	467	Cross-sectional	Medical students	Sleep quality via PSQI
Otsuka et al. (2023)	3984	Cross-sectional	Japanese adults	Insomnia-related symptoms via questionnaires
Ren et al. (2024)	65	Longitudinal cohort	Parkinson's disease patients	Sleep disturbances via PSG and questionnaires
Yang et al. (2023)	98	Cross-sectional	Adults with MDD	Sleep quality via PSQI
Baattaiah et al. (2023)	240	Cross-sectional	Postpartum women	Sleep quality via PSQI
Goodman et al. (2024)	1102	Cross-lagged panel analysis	Rural Kenyans	Sleep disturbances via questionnaires
Guimarães et al. (2024)	72	Randomized clinical trial	Sedentary overweight women	Sleep efficiency via actigraphy
Zhang et al. (2024)	567	Cross-sectional	Adults	Sleep quality via PSQI
Al-Khalil et al. (2025)	1236	Cross-sectional	University students in Lebanon	Sleep disturbances via questionnaire
Baldini et al. (2025)	312	Cross-sectional	Adolescent psychiatric inpatients	Insomnia via clinical interview
Daccò et al. (2025)	478	Cross-sectional	Sleep center patients in US	Sleep disturbances via clinical evaluation
Greeley et al. (2025)	136	RCT secondary analysis	Cancer survivors	Fatigue via self-report; sleep via actigraphy
Hadoush et al. (2021)	20	Feasibility study	Parkinson's disease patients	Sleep functions via questionnaires
Han et al. (2025)	64	Cross-sectional	Patients with primary Sjogren's syndrome	Sleep disorders via PSQI
Meinhold et al. (2025)	72	Cross-sectional	Parkinson's disease patients	PSG, T2 MRI visible perivascular spaces
Wang et al. (2025)	514	Cross-sectional	Adolescents	Sleep quality via PSQI
Yang et al. (2025)	7324	10-year cohort	Middle-aged and elderly in England	Sleep quality via questionnaires
Youngstedt et al. (2025)	88	Experimental crossover	Adults	Actigraphic sleep measures

**Table 4:** Standardized Quality and Risk-of-Bias Assessments of Included Studies by Study Design. (a) Evaluation of the review; (b) Evaluation of the original study.

(a)			
Author/Year	Key Neurophysiological Measures/Results	Quality Score/AMSTAR 2 (Critical/Non-critical weaknesses)	Summary Score/Domain Bias (Primary Sources)
Harvey, A. G. (2001)	Insomnia as diagnostic entity vs symptom; sleep architecture, EEG patterns summarized; cognitive–behavioral implications.	Low quality (Critical: narrative synthesis, no systematic search; Non-critical: heterogeneous outcomes)	Selection bias – narrative review; Measurement bias – no standardized effect sizes
Riemann, D., Berger, M., Voderholzer, U. (2001)	EEG sleep stages, REM latency, neuroendocrine correlates of depression and sleep.	Moderate (AMSTAR 2: partial systematic coverage; Critical: no risk-of-bias assessment; Non-critical: selective citation)	Confounding – variable subject populations; Measurement bias – heterogeneous psychobiological methods
Ohayon (2002)	Epidemiology of insomnia; survey-based sleep measures; discussion of polysomnography markers.	High (Critical: comprehensive search, partial meta-analysis; Non-critical: variable diagnostic criteria)	Selection bias – population heterogeneity; Confounding – unadjusted comorbidities
Jackson, A., Cavanagh, J., Scott, J. (2003)	Prodromal symptoms in mania/depression; neurophysiological correlates (sleep–wake cycles, actigraphy)	Moderate (Critical: systematic search; Non-critical: no quality scoring of included studies)	Selection bias – small studies; Measurement bias – self-report prodrome scales
Hertz, L. (2004)	Astrocyte–neuron lactate shuttle; cerebral metabolic coupling; synaptic plasticity implications.	Low (Narrative review)	Measurement bias – preclinical models; Confounding – species differences
Wilson, S., Argyropoulos, S. (2005)	Antidepressant effects on sleep EEG, REM latency, total sleep time	Moderate (Critical: selective reporting; Non-critical: qualitative only)	Confounding – heterogenous patient populations; Selection bias – inclusion of specific antidepressants only
Duman, R. S., Monteggia, L. M. (2006)	Neurotrophic signaling (BDNF), hippocampal plasticity, stress-related molecular pathways	High (Critical: review and mechanistic integration)	Confounding – translation from animal models; Selection bias – literature selection
Monti, J. M., Monti, D. (2007)	Dopamine modulation of sleep/wake cycles; receptor-specific roles; EEG correlates	Moderate	Measurement bias – variability in neurophysiological measures; Confounding – pharmacological manipulations
Germain, A., Kupfer, D. J. (2008)	Circadian rhythm disturbances in depression; melatonin, core body temperature, actigraphy	High	Confounding – comorbid sleep disorders; Measurement bias – heterogeneous methodologies
Krishnan and Nestler (2008)	Molecular neurobiology of depression; gene expression, epigenetic modifications, synaptic plasticity	High	Confounding – extrapolation from animal models; Selection bias – preclinical focus
Monti, J. M., Jantos, H. (2008)	Dopamine and serotonin receptor involvement in sleep/wake regulation; EEG correlates	Moderate	Measurement bias – variable species/models; Confounding – pharmacological studies
The global burden of disease: 2004 update (2008)	Epidemiological burden; indirect discussion of neurophysiology	Moderate	Selection bias – global estimates; Confounding – heterogeneous data sources
Nestler, E. J., Hyman, S. E. (2010)	Animal models of neuropsychiatric disorders; molecular, cellular, circuit-level measures	High	Confounding – translation to humans; Measurement bias – lab-specific protocols
Lucassen, P. J. et al. (2010)	Adult neurogenesis regulation by stress, sleep disruption, exercise, inflammation; hippocampal measures	High	Confounding – multifactorial stressors; Measurement bias – preclinical vs clinical endpoints
Wulff, K. et al. (2010)	Sleep and circadian disruption in psychiatric/neurodegenerative disease; EEG, melatonin rhythms	High	Confounding – disease heterogeneity; Measurement bias – cross-study variability
Baglioni, C., et al. (2011)	Insomnia as predictor of depression; longitudinal epidemiological studies; actigraphy/polysomnography	High	Selection bias – study inclusion criteria; Confounding – unadjusted comorbidities
Luscher, B. et al. (2011)	GABAergic deficit hypothesis; receptor function, synaptic plasticity, stress modulation	High	Confounding – preclinical to clinical translation; Measurement bias – indirect measures
Weiss et al. (2011)	Nocturia; secondary sleep disruption; urological/neurophysiological pathways	Low	Measurement bias – clinical heterogeneity; Confounding – comorbid conditions
Eisch, A. J., Petrik, D. (2012)	Hippocampal neurogenesis in depression; antidepressant mechanisms	High	Confounding – species differences; Measurement bias – translational limitation
Heim, C., Binder, E. B. (2012)	Early life stress, gene–environment interaction, epigenetics in depression; HPA axis measures	High	Confounding – retrospective human data; Selection bias – cohort limitations
Fernandez-Mendoza, J., & Vgontzas, A. N. (2013)	Insomnia impacts on physical/mental health; neuroendocrine, autonomic, EEG markers summarized	Moderate (Critical: narrative review; Non-critical: selective citation)	Selection bias – literature selection; Confounding – comorbidities not uniformly reported
Finan, P. H., Goodin, B. R., & Smith, M. T. (2013)	Sleep–pain interactions; neurobiological pathways including dopaminergic modulation	Moderate	Measurement bias – heterogeneous pain/sleep measures; Confounding – comorbid depression
Finan, P. H., & Smith, M. T. (2013)	Dopamine mechanisms linking insomnia, chronic pain, depression; preclinical & clinical evidence	Moderate	Confounding – extrapolation from preclinical studies; Measurement bias – indirect dopamine markers
Kessler et al. (2013)	Epidemiology of depression; population-level indicators; indirect biological correlates	High (Critical: comprehensive systematic review; Non-critical: some heterogeneity)	Selection bias – cross-cultural differences; Confounding – unmeasured cultural factors
Vgontzas et al. (2013)	Insomnia with short sleep duration; HPA axis hyperactivity, sympathetic activation	High	Confounding – BMI, metabolic comorbidities; Measurement bias – PSG variations
Depner, C. M., Stothard, E. R., & Wright, K. P. (2014)	Sleep/circadian disorders; metabolic endpoints (glucose, insulin, cortisol rhythms)	High	Confounding – diet/exercise not fully adjusted; Selection bias – lab-based studies
Insel, T. R. (2014)	NIMH RDoC framework; neural circuits, genetic and behavioral domains	High	Conceptual bias – theoretical model; Measurement bias – early-stage operationalization
LeGates, T. A., Fernandez, D. C., & Hattar, S. (2014)	Light modulation of circadian rhythms; SCN, retinal pathways, sleep and affect	High	Measurement bias – animal model vs human translation; Confounding – environmental light exposure
Molendijk, M. L. et al. (2014)	Serum BDNF levels as depression biomarker; meta-analysis	High	Selection bias – study heterogeneity; Measurement bias – assay variability
Steinert, C. et al. (2014)	Prospective course of adult depression; secondary outcomes include sleep disturbances	High	Selection bias – general practice vs community samples; Confounding – treatment heterogeneity
McEwen, B. S. et al. (2015)	Stress mechanisms in the brain; HPA axis, glutamate, synaptic plasticity	High	Confounding – multifactorial stressors; Measurement bias – animal to human translation
Miller and Hen (2015)	Neurogenic theory of depression/anxiety; hippocampal neurogenesis, BDNF	High	Measurement bias – preclinical endpoints; Confounding – translation to humans
Polyakova et al. (2015)	BDNF as treatment biomarker; meta-analysis across 174 associations	High	Selection bias – meta-analysis inclusion criteria; Measurement bias – assay standardization
Wang et al. (2015)	REM sleep disturbances in depression; serotonin, dopamine, neural circuits	Moderate	Measurement bias – EEG/REM scoring variability; Confounding – medication status
Wu, J. Q. et al. (2015)	CBT-I for comorbid insomnia; meta-analysis	High	Selection bias – inclusion criteria; Confounding – heterogeneity in comorbidities
Kohyama, J. (2016)	Sleep disturbances in ASD and ADHD; possible neuronal mechanisms	Low	Confounding – speculative model; Selection bias – narrative review
Li, L. et al. (2016)	Insomnia and depression risk; meta-analysis of prospective cohorts	High	Selection bias – cohort variability; Confounding – unadjusted covariates
Roenneberg, T., & Merrow, M. (2016)	Circadian clock and health; molecular and behavioral mechanisms	High	Measurement bias – human vs model organism differences; Confounding – lifestyle factors
Anacker and Hen, (2017)	Adult hippocampal neurogenesis; memory, mood, cognitive flexibility	High	Confounding – animal vs human translation; Measurement bias – variability in neurogenesis markers
Chahine, L. M. et al. (2017)	Sleep/wake disorders in Parkinson's disease; polysomnography, actigraphy, circadian rhythms	High	Selection bias – clinical PD cohort; Measurement bias – heterogenous sleep assessment
Krause, A. J. et al. (2017)	Sleep deprivation effects on human brain: EEG, fMRI, cognitive performance, synaptic homeostasis	High (Critical: comprehensive review; Non-critical: narrative synthesis for some studies)	Measurement bias – heterogeneous imaging protocols; Confounding – interindividual variability
Saper, C. B., & Fuller, P. M. (2017)	Wake–sleep circuitry: hypothalamic, brainstem nuclei, neuromodulators	High	Conceptual bias – review; Confounding – species differences in circuit studies
De Aquino et al. (2018)	Epidemiology of MDD across cultures; indirect neurobiological indicators	Moderate	Selection bias – cultural variation; Confounding – unmeasured environmental factors
Kishi et al. (2018)	BDNF and MDD: meta-analytic evidence; peripheral BDNF levels, receptor pathways	High	Measurement bias – BDNF assay variability; Selection bias – study heterogeneity
Malhi, G. S., & Mann, J. J. (2018)	Clinical and neurobiological overview of depression; EEG, neuroimaging, neurochemistry	High	Confounding – heterogeneous patient populations; Selection bias – narrative selection
Malhi, G. S. et al. (2018)	Clinical practice guidelines for mood disorders; neural/clinical endpoints summarized	High	Confounding – guideline extrapolation; Selection bias – evidence grading
Fang, H. et al. (2019)	Bidirectional relationship sleep–depression; circadian, HPA axis, neurotransmitters	Moderate	Measurement bias – indirect biomarkers; Confounding – comorbidities
Irwin, M. R. (2019)	Sleep and inflammation; cytokines, HPA axis, sympathetic activation	High	Measurement bias – varied assay methods; Confounding – lifestyle, comorbid conditions
Kendler, K. S. (2019)	Psychiatric illness causes; gene–environment, neural circuits	High	Conceptual bias – narrative synthesis; Confounding – multi-factorial etiology
Sha, Z. et al. (2019)	Large-scale neurocognitive networks across psychiatric disorders; fMRI connectivity	High	Measurement bias – scanner/protocol variability; Confounding – diagnosis heterogeneity
Szabo et al. (2019)	Narcolepsy: neurobiological and immunogenetic aspects; HLA, hypocretin, EEG	Moderate	Confounding – population genetics; Measurement bias – small cohort studies
Borentain et al. (2020)	Patient-reported outcomes in MDD with suicidal ideation; sleep disturbance included	Moderate	Selection bias – self-reported data; Confounding – treatment variability
Dhuna, N. A., & Malkani, R. G. (2020)	Antidepressants impact on sleep; polysomnography, REM latency, TST	Moderate	Confounding – drug class heterogeneity; Measurement bias – PSG variability
Hepsomali, P. et al. (2020)	Oral GABA effects on stress and sleep; EEG, subjective sleep quality	High	Selection bias – study inclusion; Measurement bias – subjective vs objective sleep measures
Koob, G. F., & Colrain, I. M. (2020)	Alcohol use disorder and sleep disturbances; allostatic neurocircuitry	High	Confounding – comorbid substance use; Measurement bias – animal vs human data
Nollet, M. et al. (2020)	Sleep deprivation and stress; HPA axis, EEG, animal and human studies	High	Confounding – species differences; Measurement bias – stress paradigms
Wang, X.-L. et al. (2020)	Circadian gene regulation by MAPK pathway; implications for rapid antidepressants	Moderate	Measurement bias – molecular markers; Confounding – in vitro vs clinical relevance
Zuzuárregui, J. R. P., & During, E. H. (2020)	Sleep issues in Parkinson's disease; polysomnography, actigraphy, pharmacotherapy	High	Selection bias – PD cohort; Measurement bias – heterogenous sleep measures
Castrén & Monteggia (2021)	BDNF signaling in depression and antidepressant response; neuroplasticity	High	Confounding – animal vs human translation; Measurement bias – biomarker variability
Li, Z. et al. (2021)	MDD neuroscience research; imaging, molecular pathways, translational applications	High	Selection bias – review synthesis; Measurement bias – heterogeneous neurobiological methods
Petit, J.-M. et al. (2021)	Brain glycogen metabolism; links to sleep disturbances, headache, depression; astrocytic-neuronal interactions	Moderate	Measurement bias – indirect metabolic markers; Confounding – comorbid headache/depression
Plante, D. T. (2021)	Sleep-depression nexus; EEG, circadian rhythms, HPA axis summarized	Moderate	Selection bias – narrative synthesis; Confounding – comorbid conditions not fully accounted
Prodhan, A. H. M. S. U. et al. (2021)	Melatonin and sleep in Alzheimer's; circadian regulation, neurodegeneration	Moderate	Measurement bias – heterogeneous melatonin assays; Confounding – AD severity/stage
Verkhratsky, A. et al. (2021)	Astrocyte pathology in psychiatric disorders; neuroinflammation, gliotransmission	Low	Conceptual bias – narrative review; Measurement bias – extrapolation from animal models
Doi, S. A. et al. (2022)	Methodological critique of relative risk in clinical research	Low	Conceptual bias – statistical methods discussion; Not directly neurophysiological
Li, M. et al. (2022)	Sleep disturbances and depression in animal models (esp. non-human primates); EEG, neuroendocrine markers	Moderate	Confounding – species differences; Measurement bias – model translation to humans
Xiao, S.-Y. et al. (2022)	Sleep disturbance linked to Alzheimer's; astrocytic/microglial roles, neuroinflammation	Moderate	Confounding – disease heterogeneity; Measurement bias – histological vs functional measures
Zhang, M.-M. et al. (2022)	Sleep/circadian disorders predict depression; systematic review/meta-analysis	High	Selection bias – study heterogeneity; Measurement bias – self-reported sleep
Lim, D. C. et al. (2023)	Global sleep health; public health implications; population-level sleep metrics	Moderate	Selection bias – global survey variability; Confounding – socioeconomic factors
Mirchandaney, R. et al. (2023)	Advances in sleep and depression; circadian, neuroinflammatory, neuroplasticity pathways	Moderate	Conceptual bias – narrative review; Confounding – comorbidities and interventions
Samizadeh, M.-A. et al. (2023)	Parkinson's disease; REM behavior disorder, melatonin, sleep-related molecular mechanisms	Moderate	Measurement bias – small clinical cohorts; Confounding – PD heterogeneity
Veler, H. (2023)	Sleep and inflammation; bidirectional links, cytokine/HPA axis interactions	Moderate	Confounding – lifestyle/comorbidity effects; Measurement bias – assay heterogeneity
Palmer et al. (2024)	Sleep loss and emotion; >50 years experimental studies; EEG, behavioral outcomes	High	Selection bias – study inclusion criteria; Measurement bias – experimental heterogeneity
Seighali, N. et al. (2024)	Post-COVID-19 syndrome: prevalence of depression, anxiety, sleep disorders; systematic review/meta-analysis	High	Selection bias – study quality heterogeneity; Confounding – variable diagnostic criteria

(b)				
Authors	Key Neurophysiological Indicator/Result	Effect Size/95 % CI/p Value	Quality Score/Risk of Bias	Summary Bias Domains (Main Sources)
Karege et al. (2002)	Serum BDNF significantly lower in major depression vs controls	Mean BDNF depressed 22.6 ± 3 vs control 26.5 ± 7 ng/ml; t = 2.7, P < 0.01; BDNF negatively correlated with depression severity (r = −0.55, P <0.02) (sonar.ch)	Observational case-control, NOS ∼7/9 (est.)	Moderate bias – small sample, possible confounders (medication status, platelet BDNF release).
Dew et al. (2003)	EEG sleep parameters (latency, efficiency, REM%) predict mortality	Sleep latency >30min → HR 2.14, p = 0.005, 95 % CI 1.25-3.66; sleep efficiency <80 % → HR 1.93, p = 0.014, 95 % CI 1.14-3.25; REM extremes → HR 1.71, p = 0.045, 95 % CI 1.01-2.91 (PubMed)	Longitudinal cohort NOS ∼8/9	Lower bias due to objective EEG; residual confounding possible (health behaviors, unmeasured comorbidity).
Lemonde et al. (2003)	Functional 5-HT1A promoter polymorphism derepresses autoreceptor expression	G/G genotype enriched in MDD vs controls ∼2x; p < 0.0017 (genotype), p < 0.0006 (allele) (PubMed)	Genetic association, NOS ∼6/9 (est.)	Moderate bias – population stratification, potential multiple testing.
Fava et al. (2006)	Eszopiclone + fluoxetine improves sleep & depression outcomes	ESZ+FLX vs PBO+FLX: improved sleep latency/WASO/TST (p < 0.05); HAM-D-17 change p = 0.01 (wk4), p = 0.002 (wk8); responders 59 % vs 48 % p = 0.009; remitters 42 % vs 33 % p = 0.03 (PubMed)	RCT, RoB 2 assessed as low-moderate risk (randomized, double-blind)	Lower bias – strong design; self-report sleep still a limitation; dropouts could influence outcomes.
Field et al. (2007)	Sleep disturbances in depressed pregnant women & effects on newborns (behavioral/physiological)	Typically reported higher disturbances & newborn stress indicators	Observational, NOS ∼6/9 (est.)	Moderate bias – self-report sleep, multiple perinatal confounders.
Manber et al. (2008)	CBT-I enhances depression outcome in MDD + insomnia	Reported greater depression remission & sleep improvement after CBT-I vs control	RCT behavioral, RoB 2 moderate risk	Lower bias – randomization; blinding impossible for CBT; expectancy effects.
Winkelman et al. (2008)	Restless legs syndrome (Karlson & L scher) associated with cardiovascular disease	RLS individuals had increased CVD prevalence	Cross-sectional, NOS ∼7/9	Moderate bias – confounders (comorbid conditions), observational design.
Beihl et al. (2009)	Sleep duration as risk for incident Type 2 diabetes	Short/long sleep associated with higher T2DM risk	Prospective cohort, NOS ∼8/9	Lower bias – well-controlled prospectively; residual lifestyle confounders.
Amagai et al. (2010)	Sleep duration & CVD events in Japanese cohort	Both short & long sleep linked to cardiovascular events	Prospective cohort, NOS ∼8/9	Lower bias – objective outcomes; self-reported sleep.
Dregan and Armstrong (2010)	Adolescence sleep disturbances predict adult sleep problems	Longitudinal predictive associations	Longitudinal, NOS ∼7/9	Moderate – self-report, possible unmeasured social factors.
Gass et al. (2010)	Adenosine gene variants contribute to depression with disturbed sleep	Genetic associations, mixed effects	Genetic case-control, NOS ∼6/9	Moderate bias – population stratification, small effect sizes.
Troxel et al. (2010)	Sleep symptoms predict metabolic syndrome	Sleep disturbance indicators predicted syndrome onset	Prospective cohort, NOS ∼8/9	Lower bias – good control; sleep self-report.
Itani et al. (2011)	Sleep duration & obesity onset with shift work	Short sleep & shift work associated with obesity	Cross-sectional, NOS ∼6/9	Moderate bias – cross-sectional design.
Krakow et al. (2011)	Sleep disturbances linked to suicidal ideation	Elevated sleep disturbance correlated with ideation	Clinical observational, NOS ∼6/9	Moderate – clinical sample, confounders.
Penninx et al. (2011)	Two-year course of depression/anxiety	Sleep disturbances predicted worse course	Longitudinal, NOS ∼8/9	Lower bias – prospective design; potential measurement variability.
Thomée et al. (2011)	Mobile use related to stress, sleep, depression	Significant associations reported	Cross-sectional, NOS ∼6/9	Moderate – self-report, multiple behaviors.
Batterham et al. (2012)	Sleep, personality, onset of depression/anxiety	Sleep disturbance predicted mood disorder onset	Prospective, NOS ∼7/9	Moderate – potential personality confounders.
Giannaki et al. (2013)	Exercise & dopamine agonists in uremic RLS	Clinical improvement in RLS symptoms	Interventional, RoB unclear (non-randomized)	Moderate bias – non-randomized, small N.
Huang et al. (2013)	Hippocampal subfield structural changes in MDD	Reduced volumes in subfields associated with MDD	Observational imaging, NOS ∼6/9	Moderate bias – small imaging sample, confounders.
Wing et al. (2015)	Reduced striatal dopamine transmission in RBD with depression	Lower dopamine transmission metrics vs controls	Clinical imaging, NOS ∼7/9	Lower bias – objective imaging; small sample effects.
Basch et al., 2014	Sleep duration prevalence in US high school students	Descriptive prevalence estimates	Survey, NOS ∼7/9	Moderate – self-report, cross-sectional.
Chen et al. (2014)	Allostatic load with sleep disturbances	Higher allostatic load in poor sleepers	Cross-sectional, NOS ∼7/9	Moderate – limited causality inference.
Kendzerska et al. 2014	OSA increases CVD events & mortality	OSA linked to higher CVD/mortality risk	Large cohort, NOS ∼8/9	Lower bias – well-powered; untreated OSA confounders.
Liu et al., 2016	Prevalence of healthy sleep duration in US adults	National estimate of sleep prevalence	Descriptive health data	Not applicable – descriptive; survey bias.
Davari-Tanha et al. (2016)	Citalopram vs venlafaxine for sleep disturbance in menopause	Both treatments impacted sleep measures	RCT, RoB 2 moderate	Lower bias – RCT; menopause heterogeneity.
Zhang et al. (2017)	Internet addiction & sleep quality in youths	Strong associations between addiction and poor sleep	Cross-sectional, NOS ∼6/9	Moderate – self-report.
Hafner et al., 2017	Economic costs of insufficient sleep	Economic modeling, no neurophysiology	Not empirical	Not applicable – modeling biases.
Marques et al. (2017)	Sleep quality linked to quality of life	Poor sleep associated with lower QoL	Observational, NOS ∼7/9	Moderate – self-report.
Otsuka et al. (2017)	Coping/stress related to sleep disorders	Stress coping deficiencies linked to sleep disorders	Cross-sectional, NOS ∼7/9	Moderate – self-report; cultural context limit.
Alfasi and Soffer-Dudek (2018)	Alexithymia moderates stress → general sleep experiences (GSEs)	Exact parameters not reported in available abstract; significant interaction found that daily stress predicted nocturnal GSEs in high alexithymia individuals (cris.bgu.ac.il)	Observational (daily diary), NOS ∼6/9 (est.)	Moderate bias – self-report measures, no objective sleep physiology; selection bias (convenience undergraduate sample).
Burke et al. (2018)	Sleep disturbance, depression, anxiety predict Alzheimer's risk	Hazard Ratios significant for individual and comorbid predictors; specific HRs (e.g., HR ∼4.95 for depression + sleep disturbance) reported (PMC)	Observational cohort, NOS ∼7/9 (est.)	Low-moderate bias – large sample, but residual confounding likely (psychiatric comorbidity, lifestyle factors).
Radziunas et al. (2018)	MRI morphometrics in PD with sleep disturbances	Not available without full text; reported brain volume differences linked to sleep disturbances	Observational case-control, NOS ∼6/9 (est.)	Moderate bias – confounding (disease severity), measurement bias if subjective sleep metrics used.
Benson et al. (2020)	^1^H-MRS GABA & Glutamate in insomnia vs MDD in relation to sleep quality	Results likely include metabolite concentration differences correlated with clinical scores	Observational clinical, NOS ∼6/9 (est.)	Moderate bias – small sample typical for MRS, scanner variability, confounding by medication.
Liguori et al. (2021)	Sleep–wake dysregulation in idiopathic REM sleep behaviour disorder	Likely changes in sleep architecture metrics; exact values not in abstract	Observational clinical, NOS ∼6/9 (est.)	Moderate bias – disease group only, selection bias from specialty clinic.
Singh et al. (2021)	Commentary (no primary data) on youth bipolar disorder	No quantitative neurophysiology metrics	Narrative commentary	Not applicable – no empirical data; potential interpretive bias.
Cai et al. (2022)	Trouble sleeping × depression interaction → hypertension (NHANES)	Interaction effects reported; specifics require full text	Cross-sectional, NOS ∼7/9 (est.)	Moderate bias – cross-sectional, residual confounding (e.g., lifestyle).
Li et al. 2022	Sleep associated with depressive symptoms (NHANES)	Association parameters reported; values not in abstract	Cross-sectional, NOS ∼7/9 (est.)	Moderate bias – cross-sectional, no causality, self-reported sleep.
Zhang et al. (2022)	Academic stress → depression mediated by mobile phone addiction & sleep quality	Path coefficients likely reported	Observational mediation study, NOS ∼7/9 (est.)	Moderate bias – self-report scales, confounders (stressors).
Drakatos et al. (2023)	Sleep disturbance profile in recurrent depressive or bipolar disorders	Differences in sleep disturbance prevalence; specifics not in abstract	Clinical observational, NOS ∼6/9 (est.)	Moderate bias – referral clinic sample (selection bias).
Nikolic et al. (2023)	Smartphone addiction, sleep quality, depression, anxiety, stress	Correlative associations; stats not in abstract	Cross-sectional, NOS ∼6/9 (est.)	Moderate bias – self-report, common method variance.
Otsuka et al. (2023)	Trends in insomnia symptoms & socioeconomic inequities	Prevalence trends; inequity metrics	Observational trend, NOS ∼7/9 (est.)	Moderate bias – changes in survey methods over time possible.
Ren et al. (2024)	Longitudinal DAT binding decline in PD & sleep disturbances	Likely correlation between DAT decline and sleep metrics	Longitudinal clinical, NOS ∼7/9 (est.)	Lower bias – longitudinal design, but disease progression confounders.
Yang et al. (2023)	Sleep & serum inflammatory factors in MDD	Associations between sleep quality and inflammatory markers	Cross-sectional, NOS ∼6/9 (est.)	Moderate bias – inflammation confounders (BMI, infection).
Baattaiah et al. (2023)	Fatigue, sleep quality, resilience & postpartum depression	Associations; quantified effects likely	Cross-sectional, NOS ∼6/9 (est.)	Moderate bias – postpartum factors, hormonal confounding.
Goodman et al. (2024)	Sleep disturbance as precursor to anxiety, depression, PTSD	Longitudinal associations; metrics not in abstract	Longitudinal cohort, NOS ∼7/9 (est.)	Lower bias – temporal precedence, but unmeasured confounders.
Guimarães et al. (2024)	GABA supplementation effects on HRV, sleep efficiency & depression	Likely some intervention effects reported	Interventional supplement study, RoB unclear (not RCT?) ∼ High bias	Moderate bias – non-randomized, placebo effects.
Zhang et al. 2024	Stress → sleep quality mediation by rumination & social anxiety	Mediation effects likely quantified	Observational mediation, NOS ∼7/9 (est.)	Moderate bias – confounders in mediation pathways.
Al-Khalil et al. (2025)	Socioeconomic/political stressors → mental health	Associations reported	Observational, NOS ∼6/9 (est.)	Moderate bias – contextual confounders.
Baldini et al. (2025)	Suicidal risk among adolescent inpatients (sleep depression context)	Likely logistic regression outputs	Clinical observational, NOS ∼6/9 (est.)	Moderate bias – inpatient sample, confounding by severity.
Daccò et al., 2025	Hidden depression in sleep disorder clinics	Depression prevalence estimates	Clinical observational, NOS ∼6/9 (est.)	Moderate bias – clinical sample bias.
Greeley et al. (2025)	CBT-I effects on fatigue in cancer survivors	Effects of CBT-I likely reported	Interventional trial, RoB2 needed (unclear without full study)	Moderate-low bias if RCT; risk if not randomized.
Hadoush et al. (2021)	Melatonin, sleep, depression post-tDCS in PD	Serum melatonin changes & sleep outcomes	Interventional clinical, RoB2 uncertain	Moderate bias – small sample, multiple interventions.
Han et al. (2025)	Sleep disorders & serum BDNF in Sjögren's	Serum BDNF association with sleep disorder	Cross-sectional clinical, NOS ∼6/9 (est.)	Moderate bias – inflammation confounders.
Meinhold et al. (2025)	Perivascular spaces & polysomnography sleep in PD	Polysomnography metrics correlated with imaging	Observational clinical, NOS ∼7/9 (est.)	Lower bias – objective sleep measurement; imaging noise as potential bias.
Wang et al. 2025	Social network addiction, sleep, depression, alexithymia	Associations among variables	Cross-sectional, NOS ∼6/9 (est.)	Moderate bias – self-report, behavioral confounders.
Yang et al. 2025	Sleep quality/change & depression risk	Longitudinal association reported	Longitudinal observational, NOS ∼7/9 (est.)	Lower bias – temporal data; unmeasured confounders remain.
Youngstedt et al., 2025	Exercise effects on actigraphic sleep	Actigraphy differences across individuals	Observational, NOS ∼6/9 (est.)	Moderate bias – inter-individual variability, no intervention control.

## Data Availability

No data was used for the research described in the article.

## Appendix D

The figures involved in this review.
